# Biomass-to-Methanol, Waste-to-Methanol, or PtX?—Quantifying the Sustainability of Alternative Methanol Production Pathways Using Life Cycle Assessment, Techno-Economic Analysis, and Multi-Criteria Decision Analysis

**DOI:** 10.1021/acs.est.5c16391

**Published:** 2026-07-20

**Authors:** Raoul Voss, Antonia Helf, Georg Weig, Florian Keller, Roh Pin Lee, Magnus Fröhling, Martin Gräbner

**Affiliations:** † Chair of Circular Economy and Sustainability Assessment, TUM Campus Straubing for Biotechnology and Sustainability, 9184 Technical University of Munich (TUM), Am Essigberg 3, Straubing 94315, Germany; ‡ Capgemini Engineering DE S.A.S. & Co. KG, Frankfurter Ring 81, Munich 80807, Germany; § Institute of Energy Process Engineering and Chemical Engineering, 26545 Technische Universität Bergakademie Freiberg (TUBAF), Fuchsmühlenweg 9 D, Freiberg 09599, Germany; ∥ Circular Carbon Technology KKT Freiberg, Fraunhofer Institute for Ceramic Technologies and Systems IKTS, Fuchsmühlenweg 9 D, Freiberg 09599, Germany; ⊥ Chair of Decarbonization & Transformation of the Industry, 38871 Brandenburg University of Technology Cottbus-Senftenberg (BTU), Erich-Weinert-Strasse 1, Cottbus 03046, Germany

**Keywords:** sustainable waste management, chemical recycling, recycling, gasification, pyrolysis, liquefaction, CO_2_ emissions, profitability, investments, costs

## Abstract

The sustainable production of methanol is gaining increasing global interest due to its potential to enable both defossilization and carbon circularity not only for the sustainability transformation of the chemical industry, but also for other carbon-intensive industries such as transportation and energy. This study comparatively evaluates the production of methanol via (1) Biomass-to-Methanol, (2) Waste-to-Methanol, and (3) Power-to-X (PtX) using life cycle assessment (LCA), techno-economic analysis (TEA), and multicriteria decision analysis (MCDA). Focusing on Germany, findings enable insights into technical (i.e., energy demands, product yields), environmental (i.e., impacts on climate change, terrestrial acidification, fossil depletion), and economic (i.e., investments, net present value, investment payback periods) parameters. These are presented as detailed physical results, complemented by economic metrics, and are further aggregated through expert assessment using analytic hierarchy processing. The use of alternative carbon feedstocks in the form of wood and packaging waste is observed to improve both environmental and economic performance. In contrast, PtX leads today not only to significantly more emissions but also to more costs. Study insights thus provide timely quantitative input to inform decision-making processes relating to the sustainability of alternative methanol pathways for the transformation of carbon-intensive sectors.

## Graphical abstract

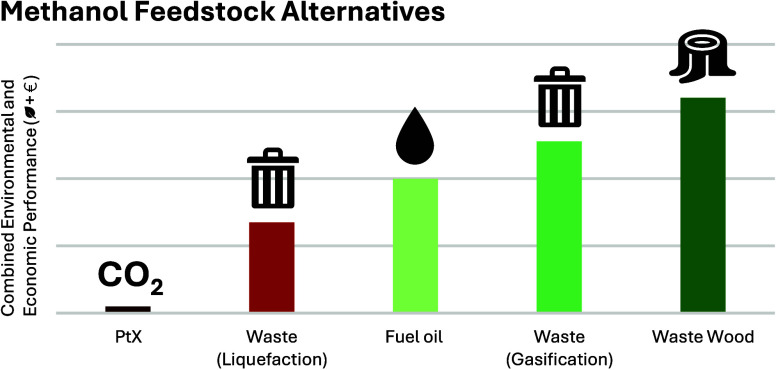

## 1 Introduction

As highlighted by the IPCC, while the effects of climate change have become increasingly evident in recent years, the implementation of abatement solutions and mitigation measures have not kept pace.^1^ Climate change abatement methods are often discussed under the umbrella term “decarbonization,” which refers to the replacement/substitution of carbon-based resources and fuels with carbon-free alternatives (e.g., coal- or gas-based energy with wind or solar power). However, for the chemical industry which is responsible for 4% of global CO_2_ emissions,^2^ decarbonization is not an option as carbon is an essential building block for chemical production, making the industry not only carbon-intensive but also carbon-dependent.

The German chemical industry—consuming 5% of Germany’s total demand of natural gas, oil, and coal—has a feedstock basis that is 87% fossil-based.^3^ Thus, to achieve climate neutrality, the goal is defossilization—the replacement of fossil feedstocks with renewable carbon sources.^4^ However, to shift away from fossil raw materials as feedstock, the pivotal question to answer is what could be a source of sustainable, nonfossil carbon for chemical production and how this could be integrated into existing process chains.

To enable the sustainability transformation of the chemical sector, methanol has been proposed as a key strategy to capitalize not only on its versatility as intermediate chemical, energy carrier, and product, but also on existing production and transportation infrastructure.^5^ However, methanol production today remains largely fossil-based. From a global perspective, natural gas reforming is the prevalent pathway of methanol production.^6,7^ In contrast, partial oxidation of heavy fuel oil is more common in Germany.^7,8^ The defossilization of methanol, i.e., sustainable production of methanol from alternative nonfossil carbon sources feedstocks is thus gaining increasing importance and is projected to play a key role in emission reduction, not only for the chemical industry but also for a variety of other carbon-intensive sectors, via enabling both defossilization and carbon circularity.^5,7,9,10^ Three sources of sustainable, nonfossil carbon that could—according to the EU commission—potentially substitute fossil-based carbon for methanol production are (1) biomass, (2) mixed waste streams, and (3) CO_2_ from point sources or atmosphere (using PtX).^11^

In recent years, biobased feedstocks have gained significant attention in the context of a bioeconomy where fossil feedstock is replaced by biomass for production.^12^ Technologically, various solutions—ranging from gasification, pyrolysis, plasma processing to anerobic digestion—exist to enable the conversion of biomass to biosyngas, followed by a subsequent conversion of the biosyngas to methanol[Fn fn1].^13^ However, while biomass-based methanol has been found to be ecologically beneficial compared to fossil-based methanol, significant technical (e.g., varying availability and composition of biofeedstocks) and economic (e.g., high capital investment) challenges have also been identified in extant literature.^15–18^ Moreover, the quantification of global biomass potential is highly challenging and also very much limited by local sustainability and economic factors.^18–20^

When looking at the utilization of waste for methanol production, an ongoing conceptual socio-political debate can be observed. In the EU, as the definition of recycling explicitly excludes fuel production, the classification of Waste-to-Methanol is thus dependent on its utilization, i.e., “recycling” when used as a chemical intermediate/product, and “recovery” when used as fuel[Fn fn2].

The literature has highlighted how the production of methanol from waste streams could—via chemical recycling[Fn fn3] in the form of waste gasification or waste pyrolysis in combination with partial oxidation and/or reforming processes^22–24^ —be used complementarily and not competitively to existing recycling pathways, with the goal to divert waste streams from incineration (i.e., transit from *“Waste-to-Energy”* to *“Waste-to-Products”*). However, the development of Waste-to-Methanol process chains faces technical challenges posed by the inhomogeneity, fluctuating properties, and contamination of waste streams, in addition to economic challenges especially in terms of high capital investments and the trade-off between economy-of-scale and local waste availability.^25–27^ Nevertheless, life cycle assessments (LCA) in multiple studies have highlighted the environmental benefits of waste-based methanol compared to fossil-based methanol.^22,25,26,28–34^

The utilization of CO_2_—either captured from point sources or from the atmosphere—as a carbon source for the chemical industry is part of the Power-to-X (PtX) strategy. First, renewable electricity is used for hydrogen production from water via electrolysis. Then, the renewable hydrogen is brought together with CO_2_ to synthesize all sorts of energy carriers, intermediates, and products. Key challenges of these pathways are the efficiency and capital investment for electrolysis units^35^ as well as difficulty in CO_2_ sourcing due to limited deployment of carbon capture technologies from air and point sources.^36^

In addition, the supply of significant amounts of emission-free and climate-friendly electricity is decisive for PtX pathways.^35,37^ LCA investigations have shown that compared to fossil-based methanol production, green methanol production via the PtX pathway is generally associated with significantly higher material resource demand but typically exhibits lower greenhouse gas emissions with strong dependency on the specific case.^38,39^ Please note that, in addition to hydrogen-based PtX pathways, direct electrochemical pathways exist in which methanol has been synthesized directly from CO_2_ electrolysis; however, these pathways are not focused in this study.

While there is a substantial body of literature on process-based assessments for methanol production from alternative carbon sources, existing studies tend to focus on individual aspects of this technological potential. This means they either examine a single alternative carbon source (i.e., biomass, or waste, or CO_2_)^40–49^ or adopt a single sustainability perspective (i.e., environmental or economic assessment).^41,44,45,48,49^ To date, there is a lack of comparative sustainability assessments that cover all three pathways for methanol production in contrast to the current state-of-the-art fossil-based methanol production under similar framework conditions to support a direct comparison of both associated environmental and economic impacts. Furthermore, the integration of multiple, potentially contradictory impact results across environmental and economic dimensions—such as through indicator weighting approaches—remains underrepresented in the literature, with only one study identified that focused on methanol production from landfill gas.^47^ Insights from a LCA study by Keller et al.^14^—which utilized a comparative LCA approach to investigate global warming potential, fossil resource depletion, and acidification potential associated with the use of fossil, biogenic, waste- and CO_2_-based pathways for olefin production in Germany—emphasized the value of such a comparative approach for multiple chemical production pathways. In their study, they found that while renewable feedstocks are generally associated with lower global warming potential and fossil resource depletion compared to fossil feedstocks, the impacts of the emission footprint of the power grid, especially for waste- and CO_2_-based pathways, can be significant.

To address the gap in the extant literature and support a holistic evaluation of the sustainability of alternative pathways for the sustainable production of methanol, this study utilizes a case analysis approach focusing on the German chemical industry to investigate different carbon-containing feedstocks systematically and comparatively for methanol production. Specifically, using LCA, three environmental impacts (i.e., impacts on climate change, terrestrial acidification, and fossil depletion) are quantified. Additionally, applying techno-economic analysis (TEA), economic impacts (i.e., total capital investments, net present value, and investment payback periods) are identified. Finally, implementing multicriteria-decision analysis (MCDA) by means of analytic hierarchy processing (AHP) involving experts’ assessments, the results are aggregated into a single indicator to facilitate decision-making processes in industrial, scientific, and political realms.

This paper is structured as follows: Section 2 provides an overview of the assessment methodology and the employed framework assumptions with regards to LCA, TEA as well as AHP. Subsequently, Section 3 presents and discusses the results of the analyses, considering the specific study context and the resulting limitations. Additionally, it provides a conclusive evaluation of this research, along with a perspective of its relevance and contribution to decision-making by policymakers and industry actors.

## 2 Materials and Methods

### 2.1 Study Setup

To identify a promising sustainable pathway for a defossilized chemical industry, representatives of each concept are included. The investigation comparatively assesses the following five different pathways for methanol production (cf. Figure 1), namely:

- Reference Case: State-of-the-Art fossil-based methanol production in Germany today.
  - Pathway 1 (PI): Partial oxidation of fossil heavy fuel oil (HFO).
- Biomass-to-Methanol: Utilization of alternative biobased feedstock (i.e., second-generation biomass such as agricultural residues and forestry waste) for methanol production.
  - Pathway 2 (PII): Wood pellets—generated from waste wood—are valorized by means of a gasification process to produce syngas which is subsequently synthesized to methanol.
- Waste-to-Methanol: Utilization of lightweight packaging waste (LWP) which is separately collected under the German dual system as alternative carbon feedstock. Today, following the separation of pure plastic streams in material recovery facilities (MRFs) for mechanical recycling, the remaining mixed plastics streams and sorting residues which cannot be mechanically recycled are incinerated for energy recovery. These waste streams thus exhibit a significant feedstock potential for methanol production via chemical recycling.^50,51^
  - Pathway 3 (PIII): Valorization of nonrecyclable LWP via a gasification process to produce syngas for downstream methanol synthesis.
  - Pathway 4 (PIV): Valorization of nonrecyclable LWP via liquefaction, yielding pyrolysis oil that is then transformed into syngas via a partial oxidation process. In this case, the pyrolysis oil serves as a substitute for HFO, and the conversion of pyrolysis oil into syngas and subsequently methanol employs the same technology pathway as in the reference pathway (PI).
- PtX: Utilization of electricity and CO_2_ for hydrogen production
  - Pathway 5 (PV): Utilize CO_2_ captured from flue gas and an electrolysis unit for hydrogen production, followed by a CO_2_-based methanol synthesis.

**Figure 1:**
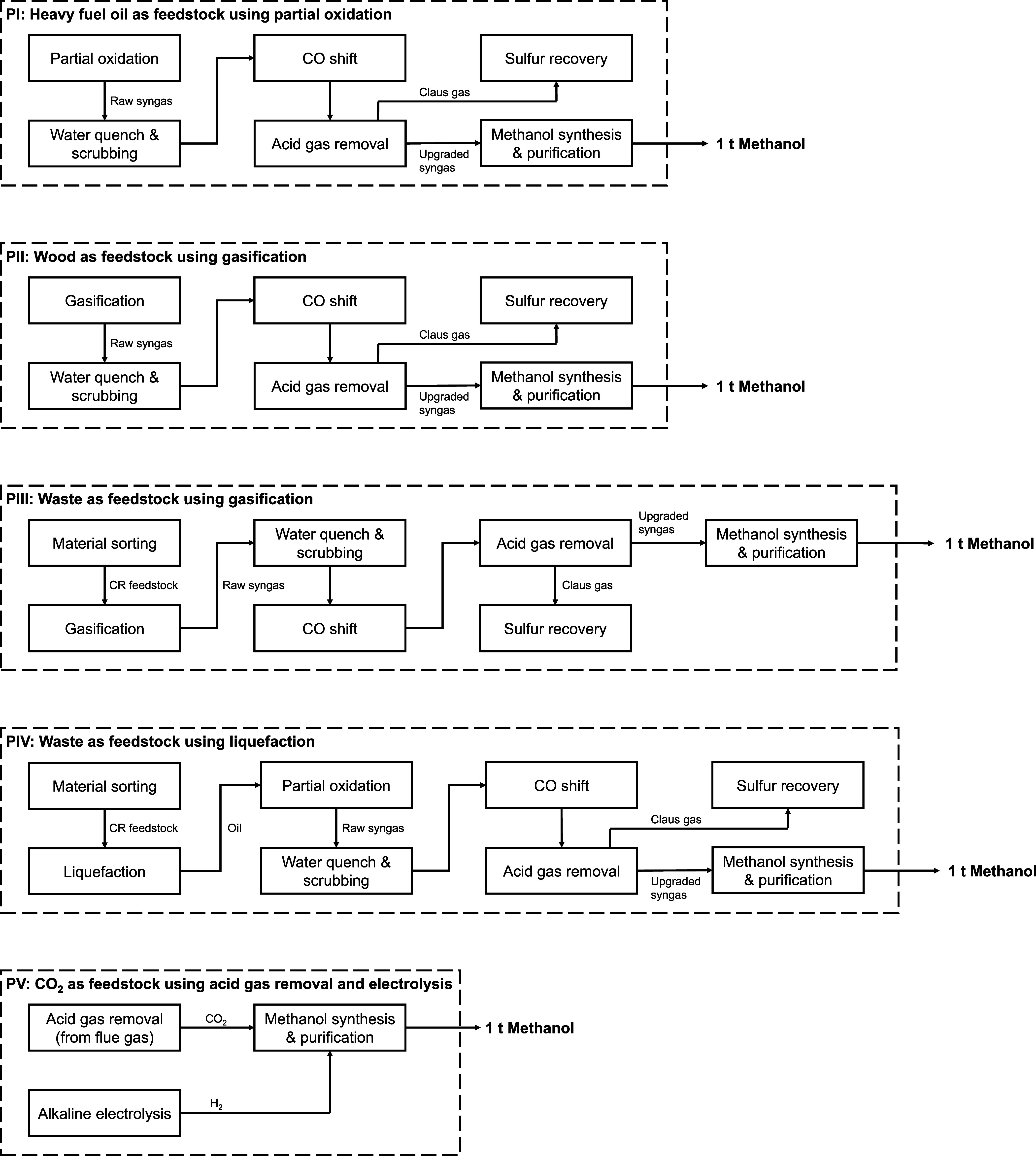
System environments for the five investigated pathways. CR: Chemical recycling.

The same methodological procedure is applied to all five pathways to ensure comparability of the generated results. The implementation of the LCA is aligned with ISOs 14040 and 14044.^52,53^ The TEA is aligned with Voss et al.^54^ Please note that the documentation of methodological assumptions, which are relevant to both TEA and LCA, such as goals and scopes (cf. Section 2.2) and process inventory assumptions (cf. Section 2.3), has been consolidated. In addition, the assumptions for the economic analysis, the AHP, and the scenario and sensitivity analyses are documented in Sections 2.4, 2.5, and 2.6, respectively.

### 2.2 Goal and Scope Definition

#### 2.2.1 Functional Unit

The functional unit for the LCA is defined as the production of virgin methanol from primary (i.e., heavy fuel oil) and secondary (i.e., waste wood, lightweight packaging waste, CO_2_) carbon feedstocks using five alternative chemical production pathways (cf. Figure 1) in tonnes of methanol production, with the reference flow defined as one tonne of virgin methanol. Conversely, the TEA uses as its functional unit an investment in a production plant that corresponds to one of the five chemical production pathways for 2025 in tonnes of production capacity. The reference flow is defined as a production capacity of 728.000 tonnes of methanol per year, aligned with the average capacity of fossil-based methanol production plants.^8^

#### 2.2.2 System Boundaries

The geographical scope of the assessment is set to Germany. Accordingly, environmental footprints, economic prizes, and assessment framework assumptions (e.g., energy grid mix) are based on values representative of Germany. Note that despite the focus on Germany, obtained results could be transferable to other geographical regions after careful evaluation and/or adjustment of all assessment conditions and taken assumptions. This includes adjustments in the assumed energy mix or the adjustment of the fossil reference production pathway for methanol. A distinctive feature of Germany as the geographical reference is that oil is the dominant fossil feedstock, which is why it was selected as the fossil reference route in this research. From a global perspective, as noted in Section 1, natural gas and coal are the prevailing pathways for methanol production. This difference must be considered when making general assumptions about this or other sustainable pathways in comparison with fossil-based methanol production in different countries or geographical reference regions.

The system boundaries for all investigated methanol production pathways located in Germany with no specific region defined are displayed in Figure 1. Please note that Figure 1 represents a simplified illustration of the individual process steps that are included in the system boundaries. More comprehensive details on energy and material flows are provided in Section 2.3, in Table 1, and from the main inventory assumption source for this assessment by Keller et al.^55^ The system boundaries for each production pathway extend from resource extraction to methanol production (i.e., cradle-to-gate). As all production pathways are expected to yield similar results for the use and end-of-life phases of the produced methanol due to similar virgin product quality (cf. Section 2.3), those phases are excluded from the assessment.

**Table 1:** Applied Inventory Balances for All Methanol Production Pathways Investigated Based on 1 Tonne of Methanol Output^a^

flow	unit	HFO	wood	LWP (Gasif.)	LWP (Liqu.)	CO_2_
input						
activated carbon	kg	0	0	0	0.29	0
ammonia	kg	0	0	0	0.25	0
catalyst (zeolite)	kg	0	0	0	27	0
diesel	kg	0	0	6.6	7.3	0
feedstock	t	0.77	1.8	3	3.3	1.4
fresh water	t	6.8	1.5	7.5	9.1	4.2
fuel gas	kg	0.076	7.4	4.2	0.077	0.058
operating fuel oil	kg	0	0	0	0.68	0
limestone	kg	0	0	0	40	0
low pressure steam	kWh	0	0	0	0	2600
oxygen	kg	900	870	780	840	0
electricity	kWh	270	380	500	870	1000
sodium hydroxide	kg	1.5	3.1	17	2.1	11
sulfuric acid	kg	1.1	1.1	1.4	1.3	7.7
output						
aluminum foil	kg	0	0	110	120	0
composites	kg	0	0	170	190	0
electricity	kWh	0	0	0	160	0
food cans	kg	0	0	280	310	0
low pressure steam	kWh	240	620	970	590	0
methanol	t	1	1	1	1	1
oxygen	t	0	0	0	0	1.5
paper and cardboard	kg	0	0	150	170	0
PE	kg	0	0	220	240	0
PET	kg	0	0	63	70	0
plastic containers	kg	0	0	49	54	0
plastic foil	kg	0	0	170	180	0
PP	kg	0	0	33	36	0
sulfur	kg	16	0.29	2.6	0.58	0
process emissions						
ash	kg	0	0	3.4	70	0
CO	kg	0.024	0.034	0.046	0.065	0.0077
CO_2_	t	0.97	1.92	1.15	1.36	0
dioxin	kg	0	1.5 × 10^–15^	0	1 × 10^–14^	0
NO_*x*_	kg	0.061	0.084	0	0.17	0.019
slag	kg	0	44	100	100	0
SO_2_	kg	1.6	1.1	0.15	0.09	0

a Utilized data for inventory modeling is described in Section 2.3 in detail. HFO: Heavy fuel oil. LWP: Lightweight packaging waste. PE: Polyethylene. PET: Polyethylene Terephthalate. PP: Polypropylene. Gasif.: Gasification. Liqu.: Liquefaction. All values are rounded to two significant digits.

For all production pathways, upstream impacts of supply production and downstream impacts of byproduct provision are accounted for by system expansion by substitution (i.e., displacement allocation) with a background system. The data of the background system is primarily sourced from the database integrated into the LCA modeling software “LCA for Experts” (cf. Section 2.2.5). In contrast, upstream impacts of feedstock provision are accounted for differently among the methanol production pathways investigated. Specifically, upstream impacts of HFO oil use (PI) are accounted for with background system data. In contrast, upstream impacts of waste wood (PII), LWP (PIII & PIV), and CO_2_ utilization (PV) are neglected due to the application of a zero-burden approach based on Montejo et al.,^56^ which is further discussed by Füchsl et al.^57^ and Fernandez et al.^58^ Based on this approach, previous environmental burdens of waste wood and LWP, such as collection and transportation of those waste streams are not considered as they are attributed to the primary product system generating the waste stream. However, it is accounted for that the alternative use of waste wood and LWP for methanol production will reduce emission intensive thermal treatment (i.e., waste incineration) of these waste fractions in Germany by providing credits to the pathways, reflecting the avoided emissions.^26,59^ Furthermore, a carbon emission credit is considered for the PtX pathway (PV), where CO_2_ is utilized for methanol production, reflecting the amount of CO_2_ utilized. Please note that footprints in the applied background process data refer to average process data suitable for Germany. Additionally, please note that environmental impacts associated with the manufacture, maintenance, and disposal of capital goods are excluded from the assessment, as their contribution to overall life cycle impacts is expected to be minor relative to operational impacts.

#### 2.2.3 Temporal Scope

The temporal scope of this assessment is oriented to the lifetime of the investigated methanol production facilities. As further elaborated in Section 2.4, this includes a construction period of three years, and a subsequent operational lifetime of 30 years. Environmental impacts are quantified assuming a steady-state plant operation across the full lifetime of 30 years. Economic impacts are quantified across the full lifetime as well, and investments during the construction phase are considered. The assessment assumes constant technical and economic framework conditions. Future technological changes, market changes, and changes in the background system are not explicitly considered but partly addressed through scenario and sensitivity analyses (see Section 2.6).

#### 2.2.4 Impact Assessment

For the environmental assessment, three midpoint indicators namely global warming potential (GWP), terrestrial acidification (TA), and fossil depletion (FD) are selected based on a detailed preassessment of the process inventories. Additionally, the selection is aligned with previously published studies on thermochemical conversion processes such as Voss et al.^26^ GWP is quantified for a period of 100 years (i.e., GWP100) and considers biogenic CO_2_ emissions to be climate relevant using impact assessment method of the IPCC as described in the Sixth Assessment Report.^60^ Impacts on TA and FD are quantified using the hierarchical mode of the ReCiPe2016 framework by Huijbregts.^61^

For the economic assessment, three economic indicators namely total capital investment (TCI), net present value (NPV), and dynamic payback period (DPP) are investigated based on Voss et al.^22^ as presented in eqs 1 to 5. This selection follows recommendations for meaningful investment decision indicators in chemical process engineering literature such as Peters et al.^62^ or Brennan.^63^ The TCI consists of the fixed capital investment (FCI) and the working capital (WC). Note that the collection and use of economic inventory data is documented in Section 2.4. Additionally, please note that all underlying equations for NPV and DPP cashflows in eqs 4 and 5 are detailed in Section 1 of the Supporting Information (SI) due to their extent.

1 $$\mathrm{FCI}=\sum_{\mathrm{components}}\left({\mathrm{FCI}}_{\mathrm{component}}{\left(\frac{\mathrm{capacity}}{{\mathrm{capacity}}_{\mathrm{ref}}}\right)}^{\mathrm{degression}\;\mathrm{coef}}\;\left(\frac{p\;\mathrm{index}}{{p\;\mathrm{index}}_{\mathrm{ref}}}\right)\right)$$

2 WC=FCI·workingcapitalrate

3 TCI=FCI+WC

4 $$\mathrm{TCI}=\mathrm{FCI}+\mathrm{WC}=\left(\sum {\mathrm{FCI}}_{\mathrm{component}}{\left(\frac{\mathrm{capacity}}{{\mathrm{capacity}}_{\mathrm{ref}}}\right)}^{\mathrm{degression}\;\mathrm{coef}}\left(\frac{p\;\mathrm{index}}{{p\;\mathrm{index}}_{\mathrm{ref}}}\right)\right){\left(1-\mathrm{working}\;\mathrm{capital}\;\mathrm{rate}\right)}^{-1}$$

5 $$\mathrm{NPV}=\sum_{\mathrm{project}\;\mathrm{years}}\frac{\sum \mathrm{cash}\;\mathrm{flows}}{{\left(1+\mathrm{discount}\;\mathrm{rate}\right)}^{\mathrm{current}\;\mathrm{year}}}-\mathrm{FCI}$$

6 $$\sum_{\mathrm{project}\;\mathrm{years}}^{\mathrm{DPP}}\frac{\sum \mathrm{cash}\;\mathrm{flows}}{{\left(1+\mathrm{discount}\;\mathrm{rate}\right)}^{\mathrm{current}\;\mathrm{year}}}-\mathrm{FCI}=0$$

#### 2.2.5 Software

“Aspen Plus V11.0”^64^ by Aspen Technology Inc. was applied as the main software solution for the generation of process inventory balances (i.e., mass and energy) for all methanol production pathways investigated in this research with corresponding modeling assumptions detailed in Section 2.3. Generated inventory balances are processed with the LCA software “LCA for Experts V8”^65^ (before “GaBi”) by Sphera Solutions with the inventory database version 2024 in custom LCA models to generate results on focused environmental impacts. Additionally, inventory balances combined with market inventory assumptions (cf. Section 2.4) are processed with Microsoft Excel^66^ in custom TEA models to generate results for the focused economic impacts.

### 2.3 Process Inventory Documentation

Applied detailed inventory balances for all five methanol production pathways are generated as displayed in Table 1, with underlying modeling assumptions documented below in detail. The selection of those processing routes and associated configurations are primarily based on Keller^51^ and Keller et al.^55^ Please note that assumed feedstock characteristics including elementary composition and lower heating value are provided with Section 2 of the SI.

#### 2.3.1 Heavy Fuel Oil (HFO) as Feedstock Using Partial Oxidation

The inventory balance for HFO as a feedstock as presented in Table 1 is drawn from Keller et al.^55^ Note that the process flow is displayed in Figure 1 and explained below in more detail with explanations directly drawn from Keller et al.^55^ HFO use is realized using partial oxidation in the first step. In addition to the oil feed, oxygen and high-pressure steam is introduced to the reaction to produce a raw synthesis gas primarily consisting of hydrogen and carbon monoxide. Note that the assumed partial oxidation process can also process other liquid hydrocarbons including pyrolysis oil (cf. Section 2.3.4).

After the partial oxidation process, the product raw synthesis gas is processed in a water wash for cooling as well as the removal of solid particles and water-soluble corrosive gas components. Sodium hydroxide is applied to regulate the pH value of the washing cycle and wastewater is extracted to limit the chloride loading. After gas washing decompression, phase separation is used to recover tar/oil and a product synthesis gas fraction. The recovered tar/oil is reintroduced into the previous partial oxidation process. The hydrogen–carbon monoxide ratio of the product synthesis gas is adjusted in a two-stage catalytic conversion process with two adiabatic fixed bed reactors. After the adjustment, sour gases are removed from the synthesis gas using a selective physical AGR (i.e., acid gas removal) process, applying low-temperature methanol. During the process Claus gas is separated for subsequent sulfur recovery.

Sulfur as a valuable raw material in the chemical industry is recovered from the Claus gas in a Claus process. Specifically, this process is realized using four stages, one thermal stage for partial oxidation and three adiabatic catalytic stages for sulfur production and separation. Note that for lower sulfur concentrations (i.e., < 50 vol.-%) a partial bypass flow around the thermal process stage is implemented. Additionally, for exceptionally low sulfur concentrations (<15 vol.-%) an iron chelate-based liquid phase oxidation process is assumed.

The last step of the process is represented by the synthesis and purification of methanol from the produced synthesis gas using an isothermal multitubular fixed-bed reactor. The unrecovered reactant gas is recycled due to limited single pass conversion. The purification of the product methanol is realized using distillation in a two-stage process. Note that the modeled methanol synthesis setup, which processes synthesis gas as feedstock, is also suitable for hydrogen and carbon dioxide mixtures (cf. Section 2.3.5).

#### 2.3.2 Wood as Feedstock Using Gasification

The inventory balance modeling for waste wood as a feedstock as presented in Table 1 is based on Keller et al.^55^ Specifically, a fixed-bed gasification reactor with liquid slag extraction (BGL type) is assumed. The gasification feedstock is introduced to the top of the reactor, while the gasification agent contacts the feedstock counter-currently. The reaction itself is an autothermal reaction (i.e., the process of heat is generated via partial oxidation of the feedstock itself) with no catalyst applied. A slag quencher is applied to extract mineral and metal components after they have been oxidized and molten by a ring burner. The targeted product of the process is raw synthesis gas for subsequent synthesis to methanol.

To upgrade and condition the raw synthesis gas, it needs to undergo all process steps described for raw synthesis gas from partial oxidation of HFO, namely water quench and scrubbing, CO shift, and acid gas removal including sulfur recovery (cf. Section 2.3.1). After upgrading and conditioning the synthesis gas is introduced to a methanol synthesis as described for HFO in Section 2.3.1 to produce product methanol.

#### 2.3.3 Lightweight Packaging Waste (LWP) as Feedstock Using Gasification

The inventory balance for LWP as a feedstock using gasification as presented in Table 1 is based on Keller^51^ and Cimpan et al.^50^ LWP as collected at the curbside represents a heterogeneous waste fraction containing parts that can be processed highly efficiently via conventional, mechanical recycling (i.e., pure plastic streams) and other parts which are nonrecyclable via mechanical recycling (i.e., mixed plastic waste streams and sorting residues) but which are highly suitable for gasification-based chemical recycling. To separate these two fractions, LWP requires a presorting step in a materials recovery facility (i.e., MRF) with sorting efficiencies assumed based on Keller^51^ as displayed in Section 3 of the SI. Subsequently, pure plastic streams can be introduced to the market to substitute for primary plastic materials. In contrast, mixed plastics and sorting residues are processed in a gasification process followed by synthesis gas upgrading and conditioning, and methanol synthesis. Those processes are set up similarly to the processes for waste wood as described in Section 2.3.2.

#### 2.3.4 Lightweight Packaging Waste (LWP) as Feedstock Using Liquefaction

Similar to the previously described process route, the inventory balance for LWP as a feedstock as presented in Table 1 is based on Keller^51^ and Cimpan et al.^50^ Rather than being processed via a gasification process as described in Section 2.3.3., mixed plastics and sorting residues produced in a MRF are processed in a liquefaction plant to produce an oil fraction. This is then processed using partial oxidation and subsequent methanol synthesis analog to Section 2.3.1. The investigated catalytic liquefaction concept generates the required process heat by friction using a slurry turbine. Specifically, after preconditioning (i.e., removal of remaining impurities such as metals), the feedstock is introduced into the process into circulating oil with a dispersed zeolite catalyst. From the process, heavy and solid components are extracted via sedimentation, while the light product oil for subsequent partial oxidation is extracted as vapor. Note that the solid and heavy liquefaction residues are processed in an existing waste incineration plant for disposal with subsequent deposition of mineral residues. The product oil subsequently undergoes the same process steps as for HFO use described in Section 2.3.1, namely partial oxidation, synthesis gas upgrading and conditioning, and methanol synthesis.

#### 2.3.5 CO_2_ from Flue Gas as Feedstock

The inventory balance for CO_2_ as a feedstock as presented in Table 1 is based on Keller et al.^55^ and Holst et al.^67^ As with the preceding methanol production pathway, the isothermal multitubular fixed-bed reactor concept with boiling water temperature control is applied. However, instead of using CO/H_2_ mixture (i.e., synthesis gas) as input, a CO_2_/H_2_ mixture is applied instead, which is assumed to be feasible with this setup.^55^ Input CO_2_ is assumed to be sourced in high purity from flue gas using a selective amine-based acid gas removal process. Hydrogen is provided using an alkaline water electrolysis process as part of the process route, without the introduction of external hydrogen.

### 2.4 Market Inventory Documentation

The market inventory includes (1) capital expenditures for treatment plants and (2) market prices for products and supplies. Capital expenditures for the 5 treatment pathways are documented in Section 4 of the SI. Note that estimates refer to greenfield plants, i.e., plants erected on a new site. Market prices for products and supplies are presented in Table 2 and are assumed as average market prices in Germany. All prices are expressed in 2025 euros.

**Table 2:** Footprints and Price Assumptions Per Unit, as Displayed in the Second Column^a^

flow	per	GWP [kg CO_2_-eq]	TA [kg SO_2_-eq]	FD [kg oil-eq]	€
activated carbon	kg	4.7 ^65^	0.0073 ^65^	2.3 ^65^	1.9 ^71^
aluminum foil	kg	6.7 ^65^	0.018 ^65^	2.3 ^65^	0.46 ^50^
ammonia	kg	2.6 ^65^	0.00054 ^65^	0.91 ^65^	0.5 ^71^
catalyst (zeolite)	kg	4.2 ^65^	0.025 ^65^	0 ^65^	2.8 ^72^
composites	kg	5.4 ^65^	0.015 ^65^	1.8 ^65^	0 ^50^
diesel	kg	0.47 ^65^	0.0017 ^65^	1.1 ^65^	1.5 ^73^
electricity	kWh	0.44 ^65^	0.00038 ^65^	0.14 ^65^	0.21 ^74^
feedstock: CO_2_	t	1000	0	0	–65 ^75^
feedstock: fuel oil	t	620 ^65^	0.98 ^65^	1000 ^65^	470 ^76^
feedstock: LWP	t	0	0	0	–210 ^50^
feedstock: waste wood	t	0	0	0	–160 ^77^
food cans	kg	2.2 ^65^	0.0031 ^65^	0.45 ^65^	0.19 ^50^
fresh water	t	1.3 ^65^	0.0016 ^65^	0.45 ^65^	4.6 ^78^
fuel gas	kg	0.63 ^65^	0.00039 ^65^	1.3 ^65^	0.38 ^71^
limestone	kg	0.93 ^65^	0.00014 ^65^	0.099 ^65^	N/R
low pressure steam	kWh	0.27 ^65^	0.0002 ^65^	0.076 ^65^	0.23 ^79^
methanol	t	N/R	N/R	N/R	700 ^80^
oxygen	t	100 ^65^	0.099 ^65^	33 ^65^	140 ^71^
paper and cardboard	kg	1.3 ^65^	0.0051 ^65^	0.42 ^65^	0.054 ^50^
PE	kg	1.9 ^65^	0.0033 ^65^	1.8 ^65^	0.13 ^50^
PET, plastic containers	kg	2.6 ^65^	0.0019 ^65^	1.6 ^65^	0.13 ^50^
plastic foil	kg	2.5 ^65^	0.0095 ^65^	1.9 ^65^	0.12 ^50^
PP	kg	1.9 ^65^	0.0016 ^65^	1.6 ^65^	0.13 ^50^
sodium hydroxide	kg	0.97 ^65^	0.0011 ^65^	0.32 ^65^	0.9 ^71^
sulfur	kg	0.79 ^65^	0.0012 ^65^	0.77 ^65^	0.21 ^71^
sulfuric acid	kg	0.22 ^65^	0.0028 ^65^	0.15 ^65^	0.098 ^71^

a FD: Fossil depletion. GWP: Global warming potential. TA: Terrestrial acidification. All prices are expressed in 2025 euros. Sources are indicated by superscript numbers. All values rounded to two significant digits.

Additional key assumptions include (1) labor prices, (2) labor requirements, (3) transportation prices, (4) plant construction and operating times, (5) the year of investment, (6) the discount rate for future cash flows, and (7) financing costs: Based on Stepstone,^68^ (1) labor prices in 2025 are assumed at €50.7 and €41.2 per hour for skilled and unskilled labor, respectively. Based on Peters et al.,^62^ (2) labor requirements are estimated based on the output of each individual relevant plant component as documented in Section 5 of the SI. (3) Market prices for transportation in 2025 are assumed at €0.069 per tonne per km based on UBA.^69^ (4) Plant construction and operation are assumed unified at 3 years and 30 years, respectively.^25^ (5) The year of investment, where plant construction starts, is set to 2025. (6) The discount rate for future cash flows is set to 8% based on Pratschner et al.^70^ (7) Finally, for reasons of simplification, additional costs related to financing have been excluded from the assessment. Note that additional economic framework assumptions such as inflation rates are presented in Section 6 of the SI.

### 2.5 Multi-Criteria Decision Analysis (MCDA)

Decision-making processes in a sustainability context often involve the consideration of trade-offs between socio-political, ecological, and economic impacts across multiple, potentially conflicting dimensions.^81,82^ MCDA provides structured methodologies to evaluate and rank alternatives within such decision-making contexts while also providing traceable information about the evaluation process, thus allowing for informed choices in complex and uncertain circumstances.^83^ Therefore, ecological and economic impact scores for each methanol production pathway evaluated in this study are aggregated into a single score through normalization and subsequent weighting, first by “Division by baseline” (DBB), with methanol production from fossil resources (PI) serving as the baseline technology. Second, a panel-based weighting approach step is used employing the AHP frequently used in combination with LCA due to its comparatively simple application and a high robustness.^84–88^

AHP is based on pairwise comparisons of all criteria against each other evaluating their relative importance along a numerical scale from one to nine, where one stands for an equal importance of both criteria for the decision-making context and nine for an extreme tendency toward one of the two criteria.^89^ Such comparative judgments are often obtained through the participation of stakeholders or experts, typically in panels ranging from as low as six up to 20 experts, focusing on a small but qualified panel with invested interest and expert understanding of the decision-making context.^90–95^

For this study, forty-three stakeholders and experts were invited to participate in an online panel, where they were asked to conduct 15 pairwise comparisons in total (cf. Section 7 of the SI for more details). The focused expert profile comprises chemical industry and market experts with multiple years of professional experience, working in the chemical industry, working in academia and conducting research on the chemical industry, as well as experts from closely linked areas (e.g., regulation and policy development for the chemical industry, NGOs, and interest groups closely linked to the chemical industry). The experts were approached based on their participation in the events “CO_2_-based Fuels and Chemicals Conference 2024” in Cologne, Germany, the “Carbon Capture and Utilization in Practice” at the “C.A.R.M.E.N.-Symposium 2024” in Straubing, Germany, and via the network of Chemical Cluster Bavaria, Germany. Thus, the expert panel covers central stakeholders along the methanol value chain and provides an internally consistent derivation of the AHP indicator weights, rather than an arbitrary weighting. However, a tendency to assign higher importance to environmental indicators cannot be completely excluded, as the experts’ professional interests and commitments to environmental topics are reflected in their participation in the aforementioned events.

Using the AHP online system (AHP OS) to obtain comparative judgments,^96–99^ ten participants completed the survey with one response not fulfilling the requirement of an internal consistency ratio of less than 0.1, thus leading to its exclusion. The aggregated geometric mean of the included panel responses yielded a ranking of the relative importance of the assessed indicators—shown in Table 3—to be used as weighting factors.^100^ To calculate a total weighted score (TWS) of the methanol production pathways assessed in this study, the assessment results are first normalized by DBB using the conventional fossil-based pathway as a benchmark.^84^ The resulting normalization scores are then multiplied by the respective weighting factors and summed up in a single TWS to facilitate decision making processes.^100^

**Table 3:** Analytic Hierarchy Process (AHP) Weighting Factors Expressed as Aggregated Geometric Means of the Panel Responses^a^

	GWP	TA	FD	TCI	NPV	DPP
Weighting Factor	0.223	0.073	0.139	0.234	0.136	0.195

a DPP: Dynamic payback period. FD: Fossil depletion. GWP: Global warming potential. NPV: Net present value. TA: Terrestrial acidification. TCI: Total capital investment.

### 2.6 Uncertainty Analysis, Scenario Analysis, and Sensitivity Analysis

Based on Quiroz et al.,^101^ LCA uncertainties are explored using a detailed Monte Carlo analysis. Specifically, a total of 500 model iterations is run, varying the model parameters identified as most critical according to a normal distribution, using the base-case value as the mean and a standard deviation of 20% of the base-case value. The respective parameters include for PI the heavy fuel oil footprint and the process efficiency (i.e., carbon efficiency in methanol production); for PII, PIII, and PIV the process efficiencies (i.e., carbon efficiency in methanol production and the energy and heat recovery efficiencies of alternative thermal treatment); and for PV the electricity and hydrogen input demands. Additionally, the assumed electricity and heat footprints are varied for all routes. Across the 500 model iterations, means and standard deviations of all impact categories (i.e., GWP, TA, FD) are evaluated for the five methanol production pathways.

Additionally, based on Keller et al.,^14^ the impact of changes in the reference energy system toward renewable energy in the upcoming years is investigated in greater detail using a scenario analysis. Specifically, in addition to the energy provision of electricity and thermal energy based on the current system energy mix (CS-EM), a predominantly renewable energy mix (PR-EM) and a climate neutral energy mix (CN-EM) are investigated, as further characterized in Section 8 of the SI.

Based on Voss et al.,^22^ uncertainties in economic assumptions are addressed using a sensitivity analysis, focusing on the key impact factors for the economic profitability of the investigated pathways such as methanol price, feedstock prices/gate fees, required investments, and CO_2_ certificate prices. The sensitivity analysis for TEA allows to highlight individual impacts of these assumptions, and the corresponding results are presented in detail as clear and easy to interpret tornado plots in Section 9 of the SI.

## 3 Results and Discussion

### 3.1 Environmental Impacts

Figure 2 illustrates the environmental impacts for all five methanol production pathways investigated in this study, subdivided into “process supply,” “feedstock use,” “product credit,” “process emissions,” and “residue treatment,” to highlight the central underlying mechanisms. While “process supply” covers impacts associated with the use of supplies such as fuel oil or water, “feedstock use” refers to impacts that are linked to the use of the respective feedstock. For biomass and LWP, this includes the reduction of thermal treatment (i.e., reduced negative incineration impacts as well as reduced positive impacts from grid electricity/heat substitution through thermal treatment). For CO_2_ use, it refers to the impacts associated with the electrolysis required to produce hydrogen. “Product credit” represents reduced impacts due to market product substitution with process byproducts (e.g., secondary plastics from LWP sorting), and “process emissions” and “residue treatment” cover impacts generated either in the process itself or from the thermal treatment of process residues. Results for the three investigated environmental impact categories, namely (a) global warming potential (GWP), (b) terrestrial acidification (TA), and (c) fossil depletion (FD) are presented in the following sections.

**Figure 2:**
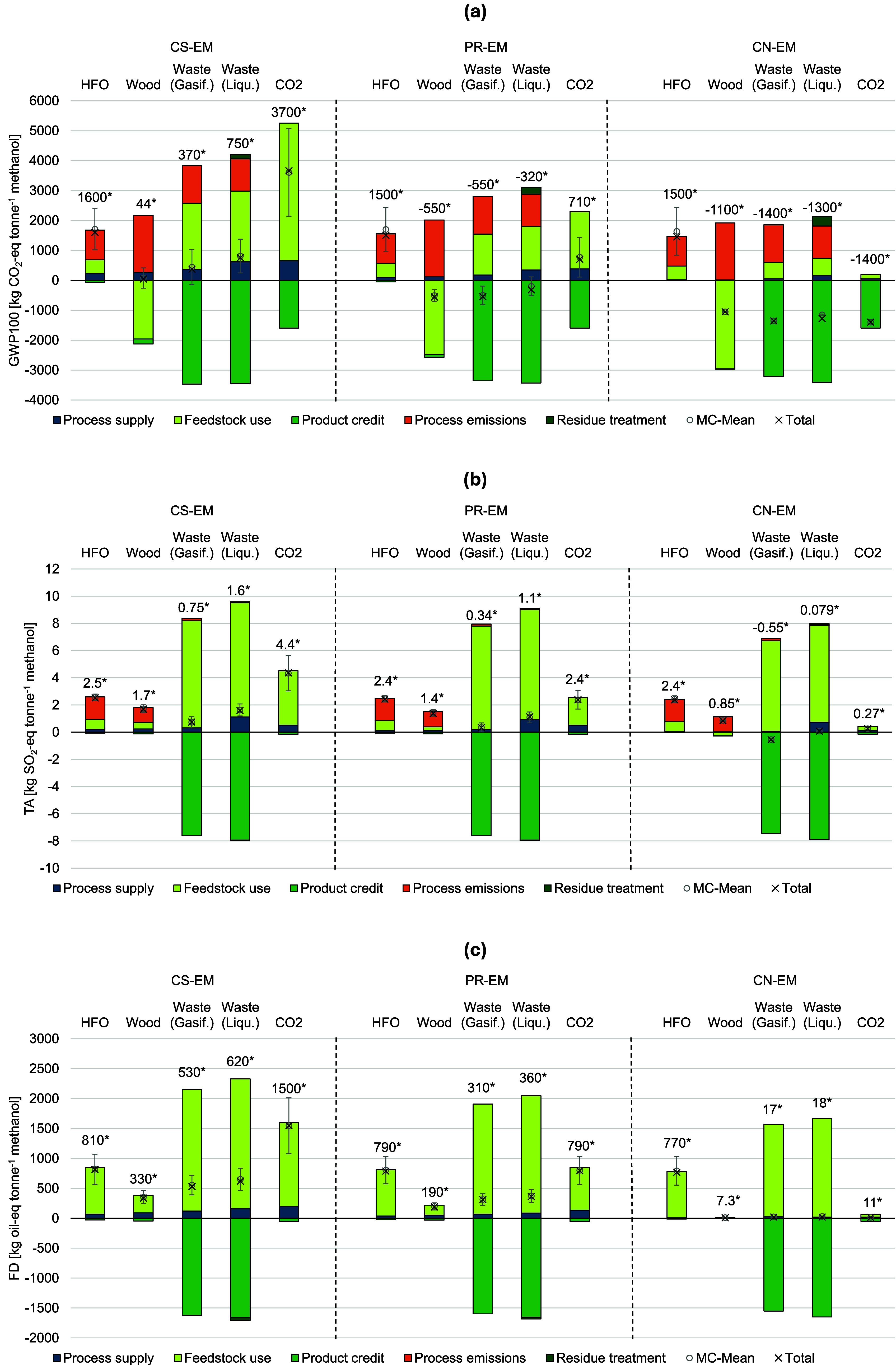
Environmental results for (a) global warming potential, (b) terrestrial acidification, and (c) fossil depletion. Monte Carlo means and standard deviation error bars displayed in gray. CN-EM: Climate neutral energy mix. CS-EM: Current system energy mix. FD: Fossil depletion. GWP: Global warming potential. HFO: Heavy fuel oil. MC: Monte Carlo. PR-EM: Predominantly renewable energy mix. TA: Terrestrial acidification. *: Value rounded to two significant digits.

#### 3.1.1 Global Warming Potential (GWP)

In the baseline energy provision scenario (i.e., CS-EM), the lowest CO_2_-eq emissions are associated with using waste wood (PII) as a feedstock at 44 kg per tonne of methanol produced. This is mainly because high process emissions on this methanol production pathway are offset by the reduction of emission-intensive waste wood incineration, leading to nearly net zero emissions. Please note that even positive impacts for biomass use (i.e., negative net emissions) are reported in the literature,^45^ which in this assessment only occurs under the assumptions of a low-carbon or climate neutral energy mix (cf. Section 3.1.4).

The second lowest CO_2_-eq emissions are produced when using LWP as a feedstock via gasification (PIII) and liquefaction (PIV) at 370 and 750 kg per tonne, respectively. On those pathways, significant emissions are produced due to the systemic reduction of energy recovery from waste (i.e., waste incineration). Specifically, in the current German energy system, energy recovery is associated with significant emission credits for substituting conventional grid energy production (i.e., ≈440 g CO_2_eq kWh^–1^). Hence, when LWP (or waste wood) is pulled out of energy recovery for methanol production (i.e., from “*Waste-to-Energy”* to “*Waste-to-Products”*), more conventional grid energy will have to be produced to make up for this shortfall. Please note that the observed emission reduction potential of methanol from waste compared to fossil-fuel methanol is well aligned with findings in the literature.^40,41^ The finding that waste-based feedstocks generally result in higher net emissions than biomass-based feedstocks is consistent with findings in the literature.^102^

Interestingly, of all five methanol production pathways investigated, the pathway associated with the highest CO_2_-eq emissions (i.e., highest GWP) is not the fossil-based methanol production pathway (PI), where significant process emissions resulted in 1600 kg per tonne methanol produced. Rather, CO_2_ use via PtX (PV) has the highest GWP at 3700 kg per tonne methanol produced (i.e., 85 times, 10 times and 5 times that of PII, PIII, and PIV, respectively). This high GWP is due to the enormous grid electricity demands associated with hydrogen provision via electrolysis (cf. Table 1). While the results for the climate-neutral energy mix scenario are well aligned with results for optimal energy provision scenarios in the literature,^42^ the other scenarios show how dependent this technological pathway is on the energy provision, leading to significantly increased emissions compared to fossil methanol feedstocks in the case of using today’s conventional grid energy.

#### 3.1.2 Terrestrial Acidification (TA)

Production of methanol from LWP is associated with the lowest SO_2_-eq at 0.75 and 1.6 kg per tonne for gasification (PIII) and liquefaction (PIV), respectively. Waste wood (PII) is associated with slightly higher emissions at 1.7 kg per tonne. Like GWP, the PtX pathway (PV) exhibits the highest emissions at 4.4 kg per tonne (i.e., between 2.75 to 6 times that of PII/PIV and PIII, respectively), even more than the conventional HFO pathway (PI) at 2.5 kg per tonne of produced methanol. Please note that the observed advantages of waste feedstock and biomass feedstock use over fossil feedstock use with respect to terrestrial acidification is consistent with existing findings in the literature, while applicable reference values could not be found for CO_2_ use.^40,49^

#### 3.1.3 Fossil Depletion (FD)

Producing methanol from waste wood is most beneficial in terms of FD at 330 kg oil-eq per tonne of produced methanol. Producing methanol from LWP is associated with higher FD at 540 and 620 kg oil-eq for gasification and liquefaction, respectively. Similar to both GWP and TA impacts, the highest FD impact is the use of CO_2_ via PtX (PV) at 1,500 kg (i.e., 4.5 times, 2.8 times and 2.4 times that of PII, PIII and PIV, respectively). This is almost double that of FD via the conventional HFO pathway (PI) at 810 kg per tonne of methanol produced. Please note that the observed advantages of waste feedstock use over fossil feedstock use with respect to fossil depletion is consistent with existing findings in the literature, while applicable reference values could not be found for Waste and CO_2_ use.^40^

Taken together, the LCA findings support conclusions in the extant literature that PtX pathways for sustainable methanol production are only environmentally competitive with fossil-based routes and alternative waste-based routes (i.e., waste wood or LWP waste) in countries where climate-neutral (i.e., 100% renewable) electricity is readily available in sufficient quantities. Based on the results, this does not apply only to global warming impacts that are well investigated in the literature but also to terrestrial acidification and fossil depletion impacts. For other countries without such ideal conditions to facilitate the environmental utilization of CO_2_ for methanol production, gasification-based production routes via Biomass-to-Methanol (i.e., for countries with access and availability of significant quantities of agricultural and/or forestry waste) and Waste-to-Methanol (i.e., theoretically applicable for all countries as carbon-containing waste is a domestic resource) are feasible and attractive options in comparison to fossil-based methanol production.

#### 3.1.4 Scenario and Uncertainty Analyses

As illustrated in Figure 2, changes in the energy mix toward 70% renewable (cf. Scenario PR-EM) or climate neutral (cf. Scenario PR-CN) lead to significant footprints reductions for all alternative methanol production pathways (PII–PV). No significant changes however are observed for HFO (PI) as this pathway is comparatively independent of external electricity and heat supplies.

The most significant GHG-intensive stage in the PtX pathway is the use of electricity for the electrolysis process (cf. Section 2.3.5). Hence, as the energy mix becomes increasingly renewable, i.e., more sustainable as in Scenario PR-EM, the environmental impacts associated with this pathway, while still higher than other alternative methanol production pathways, also decrease significantly to become better or at least on par compared with that of the HFO pathway (PI). In view of its dependence on the energy system, the sustainability of the PtX pathway thus further improves as the energy system transits toward climate neutrality.

With regards to the influence of a changing energy system on PII, PIII and PIV, as the energy system becomes increasingly renewable, the use of waste wood and LWP for energy recovery via thermal treatment (i.e., to substitute conventional grid electricity) will lead to significantly higher net emissions as credits for substituting fossil energy production will no longer be obtained (cf. Section 2.2.2). As the avoided net emissions from thermal treatment are credited to PII, PIII and PIV, which represent alternative use cases for waste wood and LWP, this not only leads to a reduction in the GWP for PII, PIII and PIV but also to a corresponding reduction in TA and FD.

Overall, the results of the scenario analysis highlight the key role of the energy system in determining the sustainability of alternative methanol production pathways. To summarize, assuming the current system energy mix in Germany, Biomass-to-Methanol (PII) and Waste-to-Methanol (PIII & PIV) are beneficial compared to the conventional methanol production route (PI) and PtX (PV). However, an energy system transition toward renewables can significantly improve the PtX route to the point where CO_2_ could eventually—with a climate neutral energy system—be on par or even outperform the use of waste wood or LWP as alternative carbon feedstock to heavy oil for methanol production. Additionally, for the Waste-to-Methanol pathway, gasification of LWP (PIII) consistently exhibits higher environmental benefits compared to liquefaction (PIV). A main reason is because solid residues from the process must be thermally treated before deposition in Germany, thus leading to additional environmental impacts for the liquefaction pathway.

Please note that the results of the uncertainty analysis, displayed in Figure 2 with Monte Carlo means and standard deviation error bars in gray, support the finding that the mode of energy provision significantly influences the final footprints of PII, PIII, PIV, and PV. The identified uncertainties are reduced when the overall impact of energy decreases due to a greener energy mix in Scenarios PR-EM and CN-EM. Additionally, uncertainties in the assessment may change the order of environmental advantageousness between the waste and waste wood routes, but that these routes become less advantageous than fossil-based or CO_2_-based methanol production seems foreclosed according to the assessment.

### 3.2 Economic Results

Figure 3 illustrates the economic results for all methanol production pathways investigated for the impact categories (a) total capital investment (TCI), (b) net present value (NPV), and (c) dynamic payback period (DPP). Please note that the detailed sensitivity analysis results, focusing on the key uncertainties and impact factors for the economic profitability of the investigated pathways, are presented in Section 9 of the SI.

**Figure 3:**
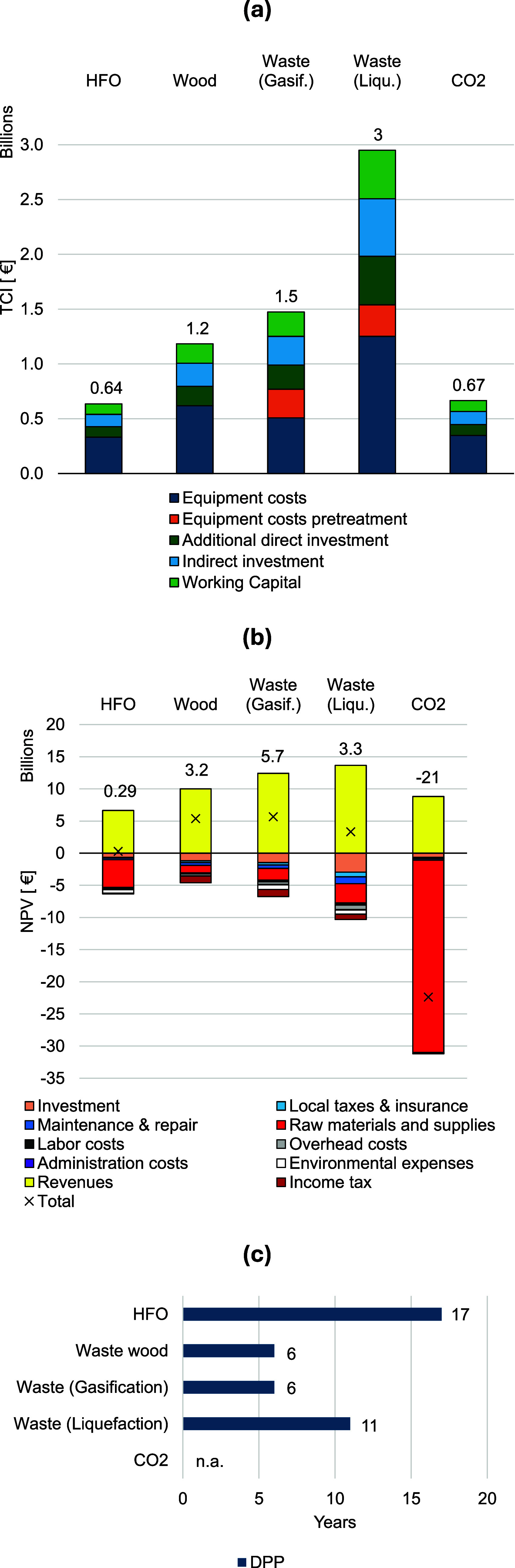
Economic results for (a) total capital investment, (b) net present value, and (c) dynamic payback period. DPP: Dynamic payback period. HFO: Heavy fuel oil. NPV: Net present value. TCI: Total capital investment.

#### 3.2.1 Total Capital Investment (TCI)

While the conventional HFO pathway (PI) is associated with the lowest TCI at 640 million €, the PtX pathway (PV)—with the least process complexity compared to all other methanol production pathways (cf. Figure 1)—requires a slightly higher TCI at 670 million €. Despite having a comparable process complexity with the conventional HFO pathway, the required TCI for Biomass-to-Methanol (PII) is almost doubled at 1.2 billion € due to low process efficiency for wood valorization via gasification, resulting in increased input capacity requirements to achieve the targeted methanol capacity (cf. Section 2.3.2).

While both pathways utilize gasification technology, at 1.5 billion €, Waste-to-Methanol via gasification of LWP (PIII) is slightly more capital intensive compared to Biomass-to-Methanol (PII) due to the necessary materials recovery step to separate pure plastic streams from mixed plastics and sorting residues (cf. Section 2.3.3). The most capital-intensive pathway is Waste-to-Methanol via liquefaction of LWP (PIV) at 3 billion € (i.e., double the TCI of gasification of LWP). This is due to the excessive costs associated with providing large liquefaction capacities (cf. Table S8 in the SI). Please note that, according to the sensitivity analysis presented in Section 9 of the SI, investment costs have a comparatively smaller impact on the overall profitability of the investigated pathways than methanol prices or feedstock and electricity costs.

#### 3.2.2 Net Present Value (NPV)

The highest NPV is obtained for the use of LWP via gasification (PIII) at 5.7 billion €. This is due to the significant revenues from processing waste which is associated with gate fee income. Additional income is also generated from selling pure plastic streams as products from material recovery (cf. Section 2.3.3). Due to a reduced overall process efficiency, the liquefaction pathway (PIV) requires a higher amount of feedstock input (i.e., more feedstock is processed/utilized to achieve the same amount of product methanol), thus leading to higher gate fee incomes and the highest revenues overall. However, the liquefaction process is also associated with the highest process complexity associated among others with higher investments, supply needs (e.g., catalyst application), and maintenance and repair costs. All these factors lead eventually to a reduced total profitability—compared to gasification—at 3.3 billion €.

Compared to Waste-to-Methanol, wood waste processing (PII) is associated with a slightly lower calculated profitability at 3.2 billion €. This is due to the reduced revenues generated via feedstock gate fees (cf. Table 2). At 290 million €, the conventional HFO pathway is still profitable but less compared to Waste-to-Methanol and Biomass-to-Methanol. This is because instead of revenues from gate fees, HFO use is assumed to incur raw material costs.

Of all five methanol production pathways, the PtX pathway (PV) is the only unprofitable pathway with significant economic losses at −21 billion €. This is due to the significant electricity requirements for this pathway. Please note that according to the conducted sensitivity analysis, even with an electricity price reduction of 50%, the PtX pathway will still lead to significant economic losses at −10 billion € (cf. Section 9 of the SI). The finding of reduced economic viability of CO_2_ use compared to fossil feedstock use is in line with results that can be found in the literature.^42^

To increase the comparability of the results with the existing body of literature, NPV results have been converted into methanol production costs as presented in Table 4. This was done by excluding methanol revenues from the calculation and dividing the resulting total costs by the total production volume across the entire plant lifetime. According to the results, conventional fossil-based methanol is produced at around 300 € tonne^–1^ methanol in alignment with the existing literature, for example, with del Pozo et al.^103^ In contrast, using waste wood or LWP as a feedstock for methanol production would lead to nearly zero production costs, due to the assumed gate fee revenues for feedstock use assumed at 160 € and 210 € for one tonne of processed feedstock (see Table 2). Excluding gate fees from the calculation would increase methanol production costs for waste wood and LWP significantly. However, except for LWP use via liquefaction they are still below HFO use. This is mainly as HFO use is associated with raw material costs, while waste wood and LWP use would at least be cost-free. Please note that costs for waste use that are below the estimated HFO use costs have also been reported, for instance, by Iaquaniello et al.^104^ Significantly higher prices are observed for the use of CO_2_ due to extensive electricity need on this route at double the market prices (see Table 2). In this study, the use of grid electricity has been assumed to follow a conservative approach and due to probable restrictions in Germany for constructing renewable energy plants in the required amounts (i.e., 7.5 TWh per year). Findings in the literature, for instance, by Meunier et al.,^105^ point to significantly lower methanol production costs, mainly because of the assumption of comparatively low electricity prices (see Section 2.3.5). On the other hand, the more complex methanol production process described by Sukdolová et al.,^106^ which is based on plasma-assisted gasification, is associated with significantly higher methanol production costs.

**Table 4:** Levelized Costs of Methanol Production^a^

	HFO	wood	waste (Gasif.)	waste (Liqu.)	CO_2_
€ tonne^–1^ methanol	270	–7.9	–21	92	1300
€ tonne^–1^ methanol (no gate fees for waste use)	-	110	230	350	-

a All values are rounded to two significant digits. All costs are expressed in euros and rounded to two significant digits.

#### 3.2.3 Dynamic Payback Period (DPP)

At 6 years, the lowest payback periods are achieved via the use of gasification to process both waste wood (PII) and LWP (PIII) for methanol production. This is due to moderate investments in combination with significant revenues from the gate fees obtained for the waste wood and LWP waste. LWP via liquefaction (PIV) is associated with a DPP of 11 years due to an increased process complexity leading to higher investments.

A payback period of 17 years is estimated for the use of HFO (PI) explained by the comparatively low incomes. Note that the payback period is not applicable to CO_2_ use as this is associated with a negative net present value, implying that the investment is not recovered over the assumed plant lifetime.

#### 3.2.4 Multicriteria Decision Analysis Results

Table 5 illustrates the aggregated scores (i.e., total weighted score/TWS) for all methanol production pathways under the various energy mix scenarios, with HFO as the benchmark. A TWS that is lower than one indicates a more favorable sustainability performance compared to the benchmark, with the lowest value being the most favorable alternative among the assessed options. In contrast, a TWS higher than one indicates a less favorable overall performance.

**Table 5:** Total Weighted Score (TWS) of Assessed Technologies^a^

	HFO	wood	waste (Gasif.)	waste (Liqu.)	CO_2_
CS-EM	1.0	0.624	0.782	1.481	N/A
PR-EM	1.0	0.505	0.602	1.274	N/A
CN-EM	1.0	0.378	0.396	1.034	N/A

a A TWS lower than 1 indicates a favorable sustainability performance compared to the benchmark. CN-EM: Climate neutral energy mix. CS-EM: Current system energy mix. HFO: Heavy fuel oil. PR-EM: Predominantly renewable energy mix.

Similar to the results for DPP, gasification-based pathways are associated with the highest combined environmental and economic performance compared to the benchmark across all scenarios. Specifically, waste wood gasification shows the highest improvement with a TWS of 0.624, 0.505, and 0.378 under CS-EM, PR-EM, and CN-EM, respectively. Similarly, Waste-to-Methanol via LWP gasification shows an improvement—albeit slightly lower than waste wood gasification—with a TWS of 0.782, 0.602, and 0.396 for the three energy scenarios, respectively. No improvement in sustainability performance is observed for Waste-to-Methanol via LWP liquefaction in any of the scenarios. Please note that for the PtX pathway, a TWS cannot be calculated due to its lack of economic feasibility (cf. Section 3.2.2). However, the fact that this pathway is not competitive in most cases from both an economic and an environmental perspective can be considered equivalent to the highest TWS across all feedstocks (cf. Sections 3.1 and 3.2). Those generated insights regarding an overall reduced performance for LWP use when liquefaction is applied and for the PtX pathway compared to fossil feedstock use contradict findings from previous studies that generally emphasize the environmental advantages of alternative feedstock use for methanol production.^45,107–109^ Using an expert-supported approach that combines six indicators leads to the conclusion that the advantage of alternative feedstock use must be decided on a case-by-case basis and with specific attention to the system framework conditions to generate consistent and robust results.

Based on the authors’ knowledge, this is the first study to integrate ecological and economic indicators in a single score using AHP-based MCDA to assess the focused methanol production pathways. Thereby, this research illustrates a simple and straightforward way to convey classification and sustainability of alternative methanol production pathways. This is especially useful in informing decision-making by nonexperts and/or decision-makers who have limited insight into technical details, or have limited resources and/or time constraints for their evaluation of such information.^83,90^ However, it is important to note that aggregating multiple indicators into a single score can lead to a loss of information granularity,^110^ and can be subjected to subjective value judgements of the panel of experts.^111^ As stated in Section 2.5, participants were primarily recruited at industry events with a focus on innovative and sustainable technologies to ensure that the expert judgments obtained in this study were based on relevant expertise. Although the indicator that received the highest weighting from the experts was an economic indicator (i.e., FCI), the possibility that the selected sample places greater emphasis on sustainability-related aspects than a differently recruited expert population cannot be entirely ruled out and should therefore be considered when interpreting the results. Another potential issue with the implemented methodology is that the ranking of alternatives based on a limited set of indicators will only reflect the performance of each alternative within the scope of that set of indicators, and this may change when other indicators are chosen.^112^

#### 3.2.5 Potential Avenues for Further Research

Despite the novel contributions to extant literature, this research could be extended in multiple ways to further increase its impact in the future. General comparisons with existing studies are provided with the study results. However, given the extensive body of literature in this field and the significant variations in LCA and TEA assumptions across studies, a more detailed meta-analysis focusing on relevant studies would be useful to further align the study results with the recent literature. A dedicated study could also aim at developing standardized guidelines on methodology and reporting for assessing methanol production pathways to increase consistency and comparability across future studies.

Future studies could explore additional or alternative impact categories in different domains. In this study, environmental and economic indicators were selected, which could be extended in subsequent studies by including social indicators, commonly used in Social LCA (S-LCA), such as employment impacts. This would enhance the comprehensiveness of the research and could improve the robustness of decisions derived from these assessments.

Furthermore, an extension potential of this research might be to investigate additional fossil reference production pathways. This could allow more generalizable conclusions regarding the preferability of alternative production pathways compared to conventional fossil-based routes. In this study, oil was selected as the reference route due to its dominance in German production facilities. From a global perspective, however, natural gas and coal are more relevant, making additional assessments along these routes a natural extension of this research in future work. Also direct electrochemical PtX pathways in which methanol is synthesized directly from CO_2_ electrolysis could be investigated as additional alternative pathways (see Section 1).

Lastly, the process-focused and largely theoretical assessment mode of this research partly isolates it from the systemic framework conditions and constraints of the real world. For instance, although they represent important (practical) criteria in the assessment of alternative methanol production pathways, feedstock availability and logistics are beyond the scope of this assessment and thus not considered. Future research could integrate such aspects in their investigations to further advance systemic studies utilizing complex system modeling approaches as applied by Zupko^113^ or Voss, Lee, and Fröhling.^27^ Such approaches could also provide relevant insights into exactly how long-range transformation strategies for the organic chemical industry in combination with the energy industry might be structured and how they could be realized. Insights and data generated in this research describe an important building block for such contributions.

#### 3.2.6 Concluding Discussion

This research indicates that, in Germany and comparable contexts, the use of gaseous carbon waste streams in PtX concepts may be hindered in its contribution to more sustainable methanol production by the high costs currently associated with electricity supply. To transition to a circular carbon economy, as discussed by Meys et al.,^114^ the smaller loop that uses solid carbon waste—before it is processed to gaseous carbon waste due to thermal treatment—might be a practical and currently more sustainable option. This insight is gained from the multidimensional sustainability assessment focusing on environmental and economic aspects that are weighted based on expert evaluation in this research. Solid carbon waste provides a reliable feedstock source and has a higher potential for emission reduction without being overly dependent on external factors such as the provision of low-cost renewable energy.

The realization of a methanol production based on solid carbon waste, however, requires significant societal and industrial efforts in countries such as Germany. This includes investments in further technological development, the large-scale implementation of methanol production systems, and strategic adjustments in waste management to ensure a waste feedstocks supply that is constant in quantity and quality. Such measures will potentially be crucial in aligning methanol production with global sustainability goals and in reaching a climate-resilient future.

## Supplementary Material



## Footnotes

a For an overview of technologies and processes for biomass conversion to methanol, please refer to Farsi^13^ and Keller et al.^14^

b Biofuels produced via the use of carbon-containing waste streams such as sewage sludge and municipal solid wastes are also referred to as third-generation biofuels based on Masum et al.^24^ and Nanda et al.^21^

c For an overview of technologies and processes for methanol production via chemical recycling, please refer to Voss et al.,^22^ Lopez et al.,^23^ and Czernik and French,^24^ respectively.

## Notes

The information contains the underlying equations for the techno-economic assessment, the chemical characteristics of the investigated feedstocks, labor requirement and investment assumptions for the investigated chemical plants, a detailed description of the conducted AHP, and detailed sensitivity analysis results (PDF)

## Abbreviations

AHP: analytic hierarchy processing
CN-EM: climate neutral energy mix
CR: chemical recycling
CS-EM: current system energy mix
DBB: division by baseline
DPP: dynamic payback period
FD: fossil depletion
GWP: global warming potential
GWP100: global warming potential for a period of 100 years
HFO: heavy fuel oil
LCA: life cycle assessment
LWP: lightweight-packaging waste
MC: Monte Carlo
MCDA: multicriteria decision analysis
NPV: net present value
PI: pathway 1
PII: pathway 2
PIII: pathway 3
PIV: pathway 4
PR-EM: predominantly renewable energy mix
PV: pathway 5
PtX: power-to-X
SI: supporting Information
TCI: total capital investment
TA: terrestrial acidification
TEA: techno-economic analysis
TWS: total weighted score
WF: weighting factor
