# Supplementary material for: Biomass-to-Methanol, Waste-to-Methanol, or PtX?—Quantifying the Sustainability of Alternative Methanol Production Pathways Using Life Cycle Assessment, Techno-Economic Analysis, and Multi-Criteria Decision Analysis
Source: Environ Sci Technol. 2026 Jul 20;60(30):20857–73. doi: 10.1021/acs.est.5c16391 (PMC13449891; doi:10.1021/acs.est.5c16391)
Supplement: Supplementary file 1 [file es5c16391_si_001.pdf]

Supplementary Information

Biomass-to-Methanol, Waste-to-Methanol, or PtX?—Quantifying the Sustainability of Alternative Methanol Production Pathways Using Life Cycle Assessment, Techno-Economic Analysis, and Multi-Criteria Decision Analysis

Raoul Voss[1,2,], Antonia Helf[3], Georg Weig[1], Florian Keller[3,4], Roh Pin Lee[5], Magnus Fröhling[1], Martin Gräbner[3,4]*

[1] Chair of Circular Economy and Sustainability Assessment, TUM Campus Straubing for Biotechnology and Sustainability, Technical University of Munich (TUM), Am Essigberg 3, Straubing, 94315, Germany

[2] Capgemini Engineering DE S.A.S. & Co. KG, Frankfurter Ring 81, Munich, 80807, Germany

[3] Institute of Energy Process Engineering and Chemical Engineering, Technische Universität Bergakademie Freiberg (TUBAF), Fuchsmühlenweg 9 D, Freiberg, 09599, Germany

[4] Circular Carbon Technology KKT Freiberg, Fraunhofer Institute for Ceramic Technologies and Systems IKTS, Fuchsmühlenweg 9 D, Freiberg, 09599, Germany

[5] Chair of Decarbonization & Transformation of the Industry, Brandenburg University of Technology Cottbus-Senftenberg (BTU), Erich-Weinert-Strasse 1, Cottbus, 03046, Germany

*Email: raoul.voss@tum.de

Content

1. Cash flow equations 3
2. Feedstock characteristics 6
3. Material recovery facility sorting efficiencies 8
4. Investment data 9
5. Labor requirements 16
6. Economic framework assumptions 18
7. AHP Documentation 19
8. Energy mix assumptions 20
9. Sensitivity analysis 21

1. Cash flow equations

Based on Voss et al.[1], Equations S.1 to S.11 are the basis for net present value and dynamic payback period calculations as introduced in Section 2.1.5. in the main document. Please note that Table S17 presents economic framework conditions that are parameters to those equations.

$$CF_1 = FCI\,(insur + lTax) \quad \text{(S.1)}$$

with

CF_1 Local taxes and insurances [€]
FCI Fixed capital investment [€]
$insur$ Insurance percentage [%]
$lTax$ Local taxes percentage [%]

$$CF_2 = FCI\,maint\,(1 + consum) \quad \text{(S.2)}$$

with

CF_2 Maintenance & repair [€]
$consum$ Consumables percentage [%]
FCI Fixed capital investment [€]
$maint$ Maintenance & repair percentage [%]

$$CF_3 = \sum_s requi_s\,price_s \quad \text{(S.3)}$$

with

CF_3 Supplies [€]
$price_s$ Price for s [€/t]
$requi_s$ Requirements s [t]s Supply [-]

$$CF_4 = (sEmploy\,sRate + usEmploy\,usRate)\,(1 + supervis) \quad \text{(S.4)}$$

with

CF_4 Labor [€]
$sEmploy$ Skilled employee hours [h]
$sRate$ Hourly rate for skilled labor [€/h]
$supervis$ Supervision percentage [%]
$usEmploy$ Unskilled employee hours [h]
$usRate$ Hourly rate for unskilled labor [€/h]

$$CF_5 = CF_4\,overhd \quad \text{(S.5)}$$

with

CF_4 Labor costs [€]
CF_5 Overhead [€]

$overhd$ Overhead percentage [%]

$$CF_6 = \frac{CF_4\, admin}{1 + supervis} \quad \text{(S.6)}$$

with

$admin$ Administration percentage [%]
CF_4 Labor costs [€]
CF_6 Administration costs [€]
$supervis$ Supervision percentage [%]

$$CF_7 = \sum_{em} outp_{em}\, costs_{em} \quad \text{(S.7)}$$

with

CF_7 Environmental expenses [€]
$costs_{em}$ Price for handling/treatment of em [€/t]
em Emission/contaminant [-]
$outp_{em}$ Output of em [t]

$$CF_8 = \sum_{sto} outp_{sto}\, dist_{sto}\, tRate \quad \text{(S.8)}$$

with

CF_8 Transportation costs [€]
$dist_{sto}$ Transportation distance for sto [km]
$outp_{sto}$ Output of sto [t]
sto Solid treatment output [-]
$tRate$ Transportation cost rate [€/km]

$$CF_9 = \sum_{prod} outp_{prod}\, price_{prod} \quad \text{(S.9)}$$

with

CF_9 Revenues from sold products [€]
$outp_{prod}$ Output of $prod$ [t]
$price_{prod}$ Selling price for $prod$ [€/t]
$prod$ Product [-]

$$tinc = \left(\sum_{k=1}^{9} CF_k - depr\right) \quad \text{(S.10)}$$

$$CF_{10} = \begin{cases} tinc\, itax & tinc > 0 \\ 0 & tinc \leq 0 \end{cases} \quad \text{(S.11)}$$

with

CF_k Annual cashflow [€]
CF_{10} Income tax [€]
$depr$ Plant depreciation [€]
$itax$ Corporate income tax rate [%]
k Cashflow index [-]

$tinc$	Taxable income [€]

2. Feedstock characteristics

Table S1: Fuel oil characteristics[2]

Elementary composition		
C	wt.-%	88.05
H	wt.-%	10.43
O	wt.-%	0.05
N	wt.-%	0.06
Cl	wt.-%	0
S	wt.-%	1.41
Sum	wt.-%	100
Heating value		
Lower heating value	MJ / kg	39.6

Table S2: Wood characteristics[3]

Moisture	wt.-%	16.00
Elementary comp.		
Ash	wt.-%, wf.	2.35
C	wt.-%, wf.	50.70
H	wt.-%, wf.	6.15
O	wt.-%, wf.	40.04
N	wt.-%, wf.	0.63
Cl	wt.-%, wf.	0.08
S	wt.-%, wf.	0.05
Sum	wt.-%, wf.	100.00
Heating value		
Lower heating value	MJ / kg	15.8

Table S3: Mixed waste stream (i.e., mixed plastics and sorting residues) characteristics[4]

Moisture	wt.-%	5.00
Elementary comp.		
Ash	wt.-%, wf.	25.62
C	wt.-%, wf.	50.49
H	wt.-%, wf.	6.77
O	wt.-%, wf.	15.31
N	wt.-%, wf.	0.78
Cl	wt.-%, wf.	0.96
S	wt.-%, wf.	0.07
Sum	wt.-%, wf.	100.00
Heating value		
Lower heating value	MJ / kg	23.12

3. Material recovery facility sorting efficiencies

Table S4: Sorting efficiencies of a materials recovery facility for lightweight packaging waste based on Keller[4] and Krell[5]. FE: Iron. N-FE: Non-iron

Category	Output yield (%)
Polyethylene (PE)	0.07
Polypropylene (PP)	0.01
Polyethylene Terephthalate (PET)	0.02
Paper & cardboard	0.05
Foils	0.06
Plastic containers	0.02
FE metals	0.09
N-FE metals	0.04
Beverage containers (Composite)	0.06
Mixed plastics (MP)	0.14
Sorting residues	0.26
Water	0.19
Sum	1.00

4. Investment data

Table S5 to Table S9 report underlying assumptions for the fixed capital investment estimations. Capital expenditures are calculated based on Equation 1 in the main document. For temporal investment indexing the Chemical Engineering Plant Cost Index (CEPCI)[6] is applied as presented in Table S10. As cost data in Table S5 to Table S9 is assumed to reflect purchased equipment costs, individual cost positions are added based on Table S11. Finally, the working capital rate is assumed at 15% (i.e., 0.15) based on Peters et al.[7].

Table S5: Capital Expenditures Heavy Fuel Oil Route. EA: Expert Assessment. All values rounded to two significant digits

Plant component	REF Price	REF Capacity	REF Capacity	REF Cap. Year	DEG Coeff.	Source	Aimed capacity
POX	140000000	180	t/h oil input	2008	0.66	[8]	64
Air separation unit	110000000	4800	t/d O2	2008	0.81	[9]	1800
Water Wash	7800000	130	m^3/s i.N. input gas	2011	0.7	[10]	64
Shift	6600000	84	m^3/s i.N. raw gas	2013	0.8	[11]	95
Rectisol	100000000	180	kg/s CO2	2008	0.53	EA	22
Claus	13000000	1700	kg/h S	2008	0.55	[12]	1300
Methanol synthesis	220000000	120	kg/s raw methanol	2011	0.86	[13]	26
Methanol upgrading	10000000	37	kg/s methanol	2011	0.32	[10]	23
Torch	790000	9.9	m^3/s i.N.	2011	0.65	[10]	3.8
Methanol storage	24000000	60000	m^3	2011	0.65	[10]	18000

Table S6: Capital Expenditures Wood Route. EA: Expert Assessment. All values rounded to two significant digits

Plant component	REF Price	REF Capacity	REF Capacity Unit	REF Capacity Year	DEG Coefficient	Source	Aimed capacity
FlexiSlag gasifier	140000000	50	t/h Wood	2018	0.68	EA	150
Feedwater & BOP Plants	20000000	140	kg/s Wood	2007	0.7	[14]	43
Air separation unit	110000000	4800	t/d O2	2008	0.81	[15]	1700
Water wash	7800000	130	m³/s i.N. input gas	2011	0.7	[10]	86
Shift	6600000	84	m³/s i.N. raw gas	2013	0.8	[11]	120
Rectisol	100000000	180	kg/s CO2	2008	0.53	EA	41
Claus	13000000	1700	kg/h S	2008	0.55	EA	24
Methanol synthesis	220000000	120	kg/s raw methanol	2011	0.86	[13]	26
Methanol upgrading	10000000	37	kg/s methanol	2011	0.32	[10]	23
Torch	790000	9.9	m³/s i.N.	2011	0.65	[10]	5.4
Methanol storage	24000000	60000	m³	2011	0.65	[10]	18000

Table S7: Capital Expenditures Lightweight Packaging Waste (Gasification) Route. EA: Expert Assessment. All values rounded to two significant digits

Plant component	REF Price	REF Capacity	REF Capacity Unit	REF Capacity	DEG Coefficient	Source	Aimed capacity
**MRF**							
Preefeeding & preconditioning	210000	75000	tonnes LWP / year	2012	1	[16]	7900000
Conditioning	1200000	75000	tonnes LWP / year	2012	1	[16]	45000000
Sorting	3600000	75000	tonnes LWP / year	2012	1	[16]	140000000
Refining	350000	75000	tonnes LWP / year	2012	1	[16]	13000000
Product handling etc.	1600000	75000	tonnes LWP / year	2012	1	[16]	59000000
**Gasification**							
FlexiSlag gasifier	140000000	50	t/h RDF	2018	0.68	EA	100
Feedwater & BOP Plants	20000000	140	kg/s RDF	2007	0.7	[14]	28
Air separation unit	110000000	4800	t/d O_2	2008	0.81	[15]	1600
Water wash	7800000	130	m³/s i.N. input gas	2011	0.7	[10]	72
Shift	6600000	84	m³/s i.N. raw gas	2013	0.8	[11]	100
Rectisol	100000000	180	kg/s CO_2	2008	0.53	EA	25
Claus	13000000	1700	kg/h S	2008	0.55	EA	210
Methanol synthesis	220000000	120	kg/s raw methanol	2011	0.86	[13]	26
Methanol upgrading	10000000	37	kg/s methanol	2011	0.32	[10]	23
Torch	790000	9.9	m³/s i.N.	2011	0.65	[10]	4.5
Methanol storage	24000000	60000	m³	2011	0.65	[10]	18000

Table S8: Capital Expenditures Lightweight Packaging Waste (Liquefaction) Route. EA: Expert Assessment. All values rounded to two significant digits

Plant component	REF Price	REF Capacity	REF Capacity Unit	REF Capacit	DEG Coef	Source	Aimed capacity
**MRF**							
Preefeeding & preconditioning	210000	75000	tonnes LWP / year	2012	1	[16]	8700000
Conditioning	1200000	75000	tonnes LWP / year	2012	1	[16]	50000000
Sorting	3600000	75000	tonnes LWP / year	2012	1	[16]	150000000
Refining	350000	75000	tonnes LWP / year	2012	1	[16]	14000000
Product handling etc.	1600000	75000	tonnes LWP / year	2012	1	[16]	65000000
**Liquefaction**							
Pyrolysis	4000000	400	l/h	2018	1	[17]	76000
POX	140000000	180	t/h oil input	2008	0.66	[8]	63
Air separation unit	110000000	4800	t/d O_2	2008	0.81	[15]	1700
Water wash	7800000	130	m^3/s i.N. input gas	2011	0.7	[10]	61
Shift	6600000	84	m^3/s i.N. raw gas	2013	0.8	[11]	92
Rectisol	100000000	180	kg/s CO_2	2008	0.53	EA	20
Liquid redox sulfur recovery	2900000	1800	kg/h S	2017	1	[18]	48
Methanol synthesis	220000000	120	kg/s raw methanol	2011	0.86	[13]	26
Methanol upgrading	10000000	37	kg/s methanol	2011	0.32	[10]	23
Torch	790000	9.9	m^3/s i.N.	2011	0.65	[10]	4.6
Methanol storage	24000000	60000	m^3	2011	0.65	[10]	18000

Table S9: Capital Expenditures Carbon Dioxide Route. EA: Expert Assessment. All values rounded to two significant digits

Plant component	REF Price	REF Capacity	REF Capacity Unit	REF Capacity Year	DEG Coefficient	Source	Aimed capacity
Electrolysis	75000000	34	kg/s H2	2021	1	[19]	4.5
Flue gas separation (Amine scrubbing)	370000	0.07	kg/s CO2	2010	0.7	[20]	34
Methanol synthesis	45000000	300	t/d	2019	0.86	[20]	2000
Torch	790000	9.9	m^3/s i.N.	2011	0.65	[10]	17
Methanol storage	24000000	60000	m^3	2011	0.65	[10]	18000

Table S10 Applied values for CEPCI-index[6]. *: Value approximated with linear regression

Year	Index	Year	Index	Year	Index
…	…	2014	576.1	2021	708.8
2008	575.4	2015	556.8	2022	816.6
2009	521.9	2016	541.7	2023	797.9
2010	550.8	2017	567.5	2024	797.6
2011	585.7	2018	603.1	2025	751.0*
2012	584.6	2019	607.5	2026	765.1*
2013	567.1	2020	569.2	…	…

Table S11: Fixed capital investment (FCI) composition based on Peters et al.[7]. All values rounded to two significant digits

Description	% of FCI
Purchased equipment	61
Instrumentation and controls	6.7
Buildings	9.5
Land	1.4
Engineering & supervision	11
Contingency	9.5

5. Labor requirements

Based on Peters et al.[7], labor requirements are estimated based on the output capacity of each individual plant component that is considered to require individual operation and maintenance staff. In this, the relation between output capacity and required employee hours (EH) is modelled via equation S.12 oriented towards Peters et al.[7].

$$Required\ EE\ per\ year = (11.198\ \ln(output\ capacity\ in\ kg\ per\ day) - 75.58) * 365 \qquad (S.12)$$

Table S12: Labor requirements heavy fuel oil. All values rounded to two significant digits

Plant component	Value (kg/day)	yes/no	Employee hours (EH/year)
Partial oxidation	4000000	yes	35000
Air separation unit	1800000	yes	31000
Water wash	6200000	yes	36000
Shift	4000000	yes	35000
Rectisol	2200000	yes	32000
Claus	31000	yes	15000
Methanol synthesis	2200000	yes	32000
Methanol upgrading	2000000	no	-
Torch	230000	no	-
Methanol storage	2000000	no	-
Sum			216000

Table S13: Labor requirements wood. All values rounded to two significant digits

Plant component	Value (kg/day)	Included	Employee hours (EH/year)
FlexiSlag gasifier	6700000	yes	37000
Feedwater & BOP Plants	6700000	no	-
Air separation unit	1700000	yes	31000
Water wash	8800000	yes	38000
Shift	6000000	yes	36000
Rectisol	2400000	yes	32000
Claus	580	no	-
Methanol synthesis	2200000	yes	32000
Methanol upgrading	2000000	yes	32000
Torch	430000	no	-
Methanol storage	2000000	no	-
Sum			238000

Table S14: Labor requirements light-weight packaging waste using gasification. All values rounded to two significant digits

Plant component	Value (kg/day)	Included	Employee hours (EH/year)
Material recovery	2400000	yes	230000
FlexiSlag gasifier	4800000	yes	35000
Feedwater & BOP Plants	4800000	no	-
Air separation unit	1600000	yes	31000
Water wash	7100000	yes	37000
Shift	4500000	yes	35000
Rectisol	2400000	yes	32000
Claus	5200	no	-
Methanol synthesis	2200000	yes	32000
Methanol upgrading	2000000	yes	32000
Torch	310000	no	-
Methanol storage	2000000	no	-
Sum			464000

Table S15: Labor requirements light-weight packaging waste using liquefaction. All values rounded to two significant digits.

Plant component	Value (kg/day)	Included	Employee hours (EH/year)
Material recovery	2700000	yes	240000
Pyrolysis	1500000	yes	31000
POX	3800000	yes	34000
Air separation unit	1700000	yes	31000
Water wash	6000000	yes	36000
Shift	3900000	yes	34000
Rectisol	2200000	yes	32000
Liquid redox sulfur recovery	1200	yes	1200
Methanol synthesis	2200000	yes	32000
Methanol upgrading	2000000	yes	32000
Torch	18000	no	-
Methanol storage	2000000	no	-
Sum			503200

Table S16 Labor requirements CO_2. All values rounded to two significant digits.

Plant component	Value (kg/day)	Included	Employee hours (EH/year)
Electrolysis	380000	yes	25000
Flue gas separation (Amine scrubbing)	3000000	yes	33000
Methanol synthesis	2000000	yes	32000
Torch	210000	no	-
Methanol storage	2000000	no	-

Sum	90000

6. Economic framework assumptions

Table S17 presents economic framework conditions that are relevant to calculate cash flows according to Section 1 of this supplementary information.

Table S17: Economic framework assumptions. The underlying values rounded to two significant digits

Assumption	Value	Source
Administration percentage [%]	25	[7]
CO_2 certificate price inflation [%]	5	[21]
Consumables percentage [%]	15	[7]
Corporate income tax rate [%]	15	[22]
Depreciation period (linear) [a]	10	[23]
Insurance percentage [%]	1	[7]
Labor cost inflation rate [%]	2.65	[24]
Local tax percentage [%]	2	[7]
Maintenance & repair percentage [%]	4	[25]
Overhead percentage [%]	50	[7]
Price inflation [%]	2	[26]
Supervision percentage [%]	15	[7]

7. AHP Documentation

Figure S1 presents the online questionnaire used for pairwise impact category comparisons by participants within the analytic hierarchy process (AHP).

Pairwise Comparison AHP-OS

Evaluation of Criteria for Assessing methanol production pathways

Pairwise Comparison Assessing methanol production pathways

15 pairwise comparison(s). Please do the pairwise comparison of all criteria. When completed, click *Check Consistency* to get the priorities.

AHP Scale: 1- Equal Importance, 3- Moderate importance, 5- Strong importance, 7- Very strong importance, 9- Extreme importance (2,4,6,8 values in-between).

With respect to *Assessing methanol production pathways*, which criterion is more important, and how much more on a scale 1 to 9?

	A - wrt Assessing methanol production pathways - or B?		Equal	How much more?
1	Global Warming Potential (GWP)	Terrestrial Acidification (TA)	1	2 3 4 5 6 7 8 9
2	Global Warming Potential (GWP)	Fossil Resource Scarcity (FRS)	1	2 3 4 5 6 7 8 9
3	Global Warming Potential (GWP)	Net Present Value (NPV)	1	2 3 4 5 6 7 8 9
4	Global Warming Potential (GWP)	Total Capital Investment (TCI)	1	2 3 4 5 6 7 8 9
5	Global Warming Potential (GWP)	Dynamic Payback Period (DPP)	1	2 3 4 5 6 7 8 9
6	Terrestrial Acidification (TA)	Fossil Resource Scarcity (FRS)	1	2 3 4 5 6 7 8 9
7	Terrestrial Acidification (TA)	Net Present Value (NPV)	1	2 3 4 5 6 7 8 9
8	Terrestrial Acidification (TA)	Total Capital Investment (TCI)	1	2 3 4 5 6 7 8 9
9	Terrestrial Acidification (TA)	Dynamic Payback Period (DPP)	1	2 3 4 5 6 7 8 9
10	Fossil Resource Scarcity (FRS)	Net Present Value (NPV)	1	2 3 4 5 6 7 8 9
11	Fossil Resource Scarcity (FRS)	Total Capital Investment (TCI)	1	2 3 4 5 6 7 8 9
12	Fossil Resource Scarcity (FRS)	Dynamic Payback Period (DPP)	1	2 3 4 5 6 7 8 9
13	Net Present Value (NPV)	Total Capital Investment (TCI)	1	2 3 4 5 6 7 8 9
14	Net Present Value (NPV)	Dynamic Payback Period (DPP)	1	2 3 4 5 6 7 8 9
15	Total Capital Investment (TCI)	Dynamic Payback Period (DPP)	1	2 3 4 5 6 7 8 9

CR = 0% Please start pairwise comparison

Calculate

AHP-OS author: Klaus D. Goepel, BPMSG. Contact Last update: Feb 26, 2022 Rev: 172

Figure S1: Online panel questionnaire

8. Energy mix assumptions

Table S18: Electricity mix assumptions oriented towards IEA[27]. CN-EM: Climate neutral energy mix. CS-EM: Current system energy mix. PR-EM: Predominantly renewable energy mix. Values for scenario "CS-EM" refer to the most current published data for 2021.

	CS-EM (%)	PR-EM (%)	CN-EM (%)
Lignite	17	0	0
Hard coal	13	0	0
Oil	1	0	0
Natural gas	16	33	0
Biofuels	8	12	0
Waste	2	0	0
Nuclear	12	0	0
Hydro	4	5	0
Solar PV	8	13	0
Wind	19	37	100
Other sources	0	0	0
Geothermal	0	1	0
**Sum**	**100**	**100**	**100**

Table S19: Thermal energy mix assumptions oriented towards Agora Energiewende[28]. CN-EM: Climate neutral energy mix. CS-EM: Current system energy mix. PR-EM: Predominantly renewable energy mix.

	CS-EM (%)	PR-EM (%)	CN-EM (%)
Electricity	0	40	100
Geothermal	0	9	0
Biomass	17	4	0
Hard coal	23	0	0
Lignite	8	0	0
Gas	43	19	0
Solar	0	14	0
Other	9	15	0
**Sum**	**100**	**100**	**100**

9. Sensitivity analysis

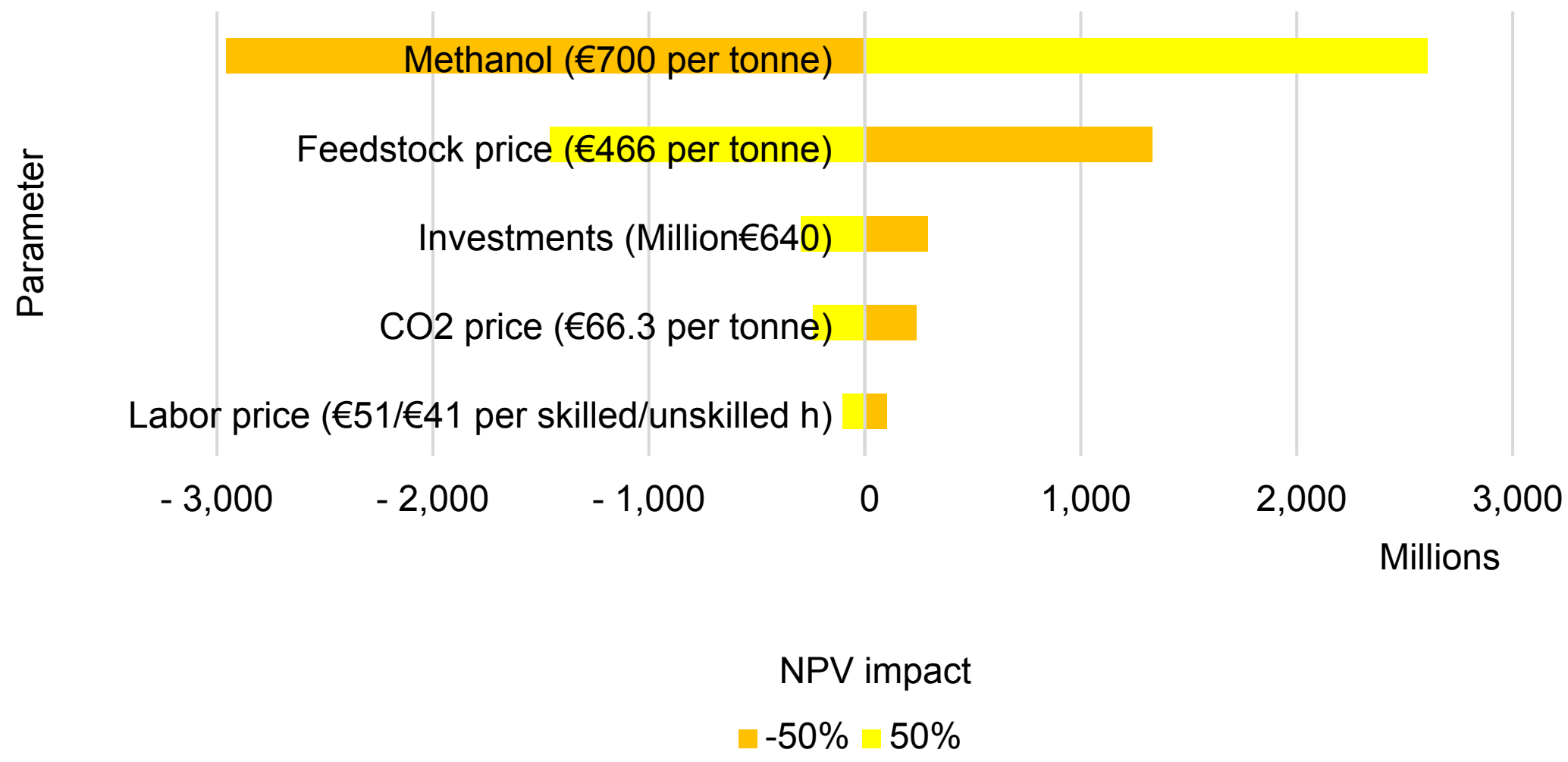

Figure S2: NPV sensitivity analysis results for oil use

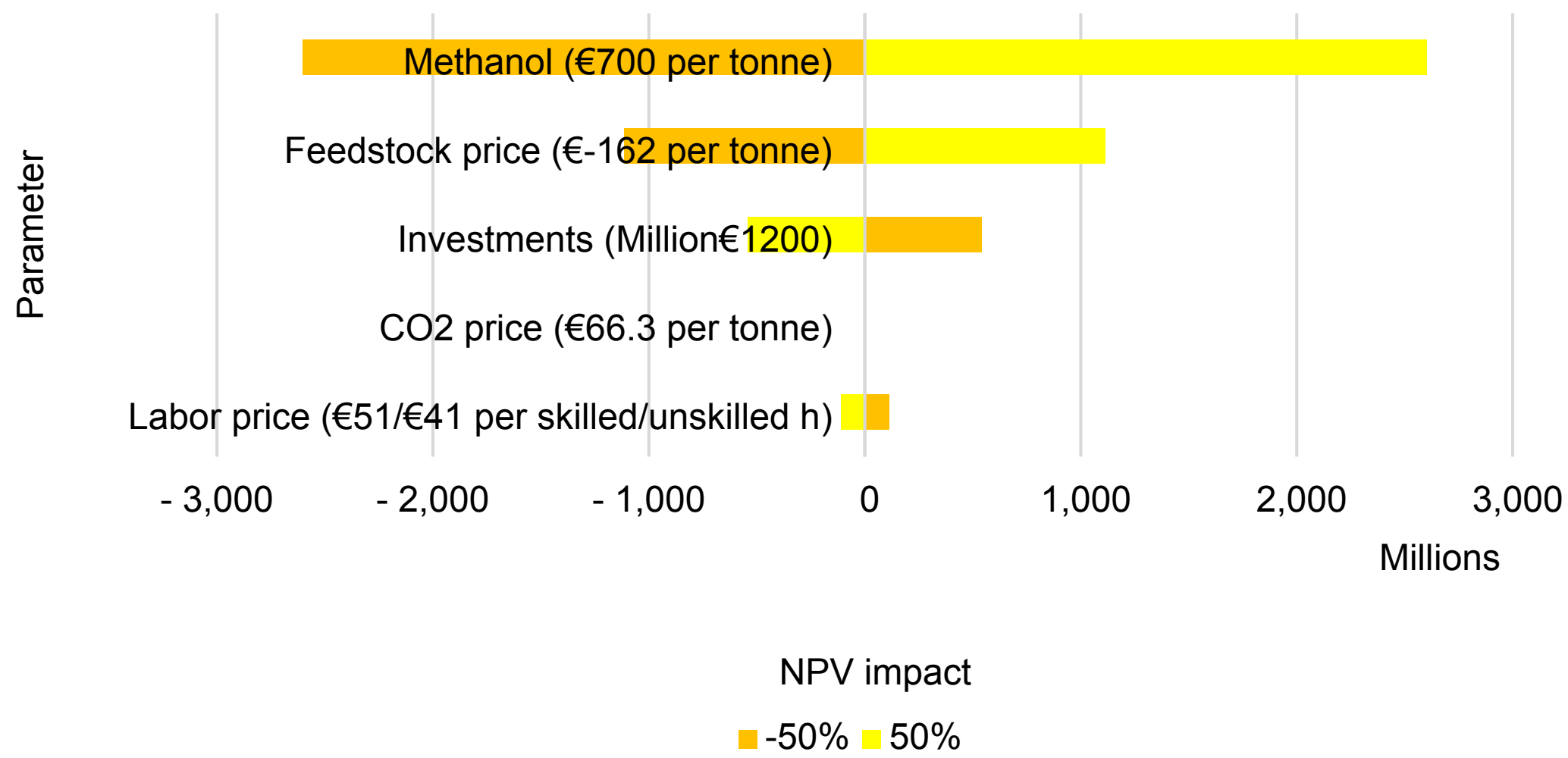

Figure S3: NPV sensitivity analysis results for wood use

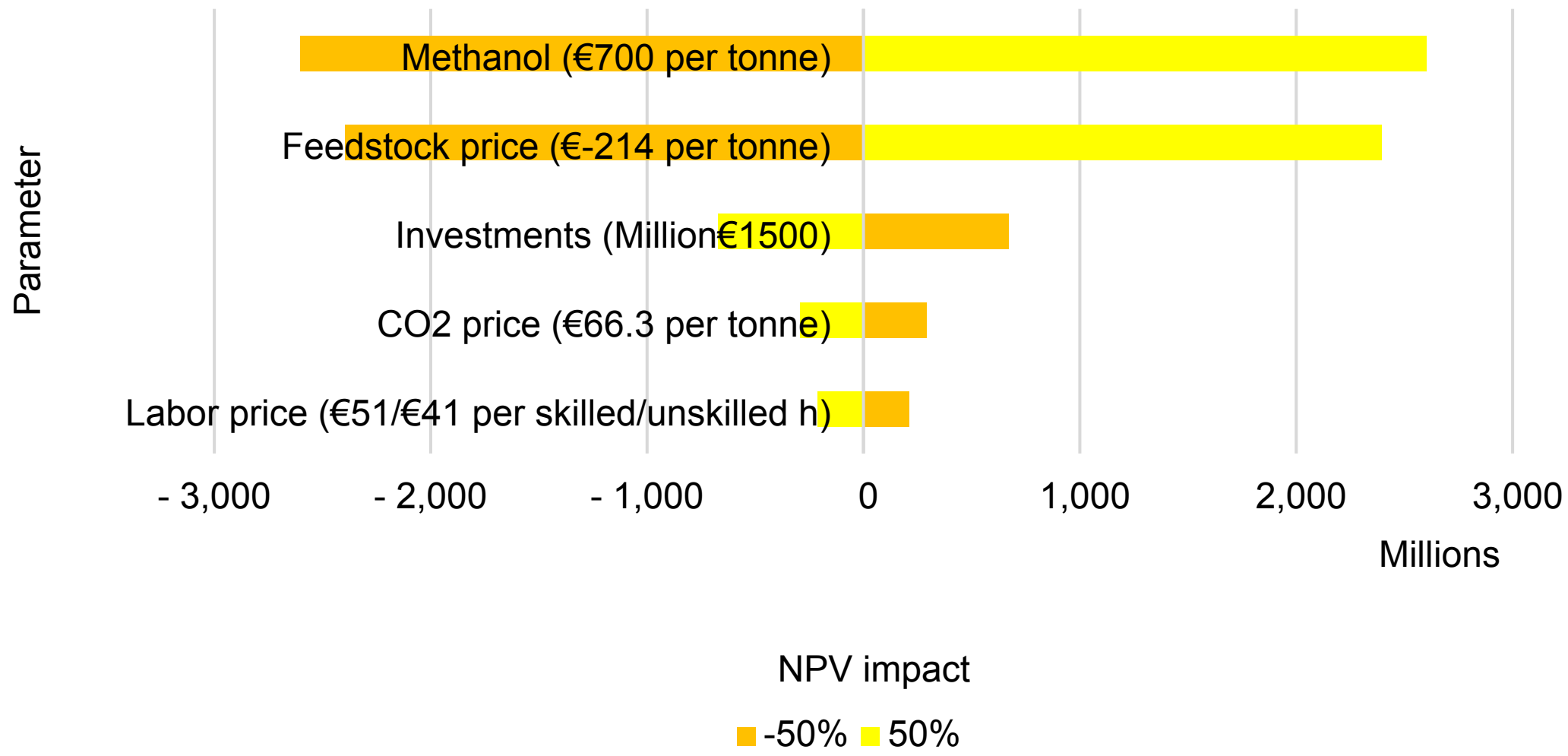

Figure S4: NPV sensitivity analysis results for light-weight packaging waste use using gasification

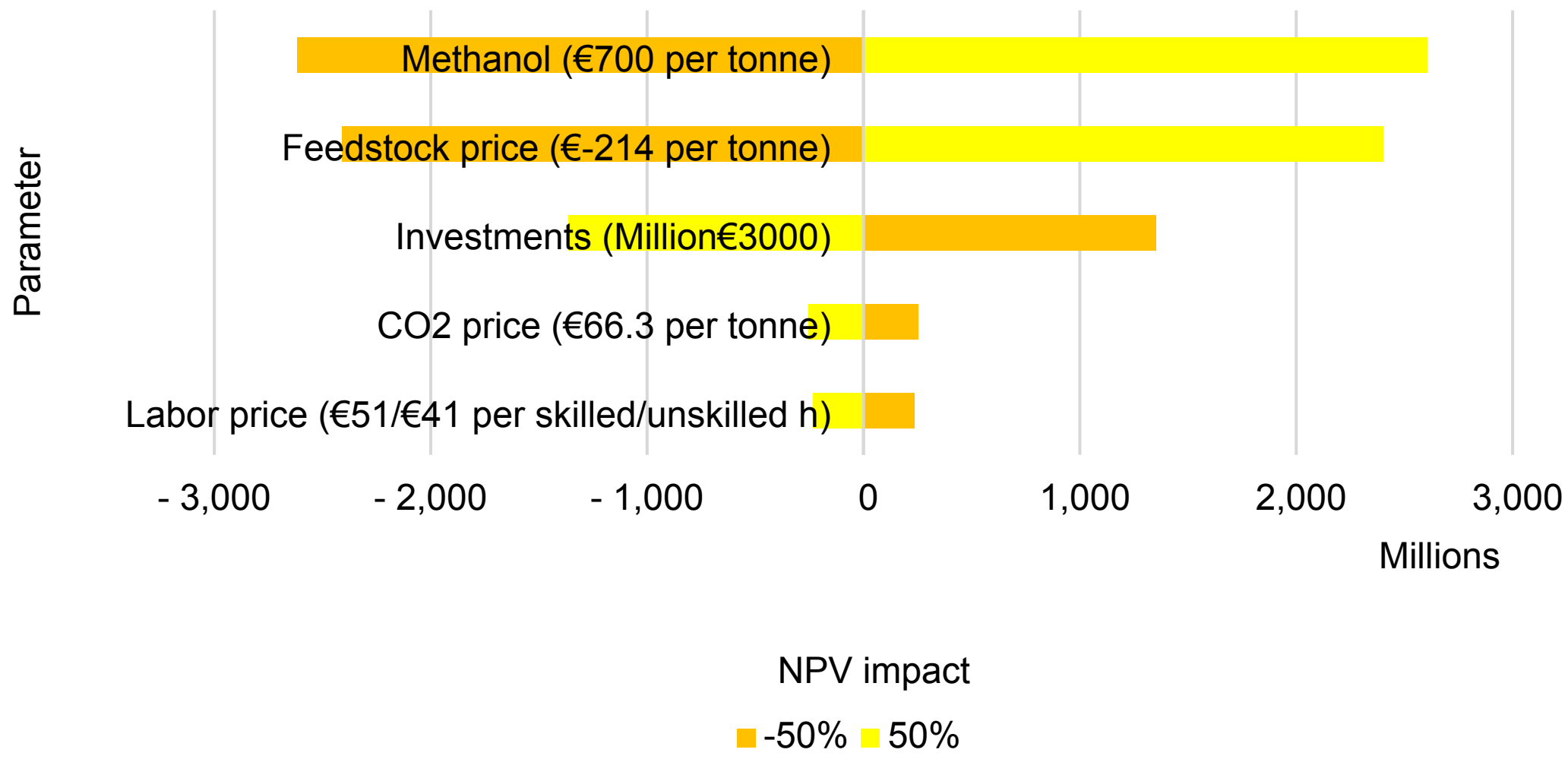

Figure S5: NPV sensitivity analysis results for light-weight packaging waste use using liquefaction

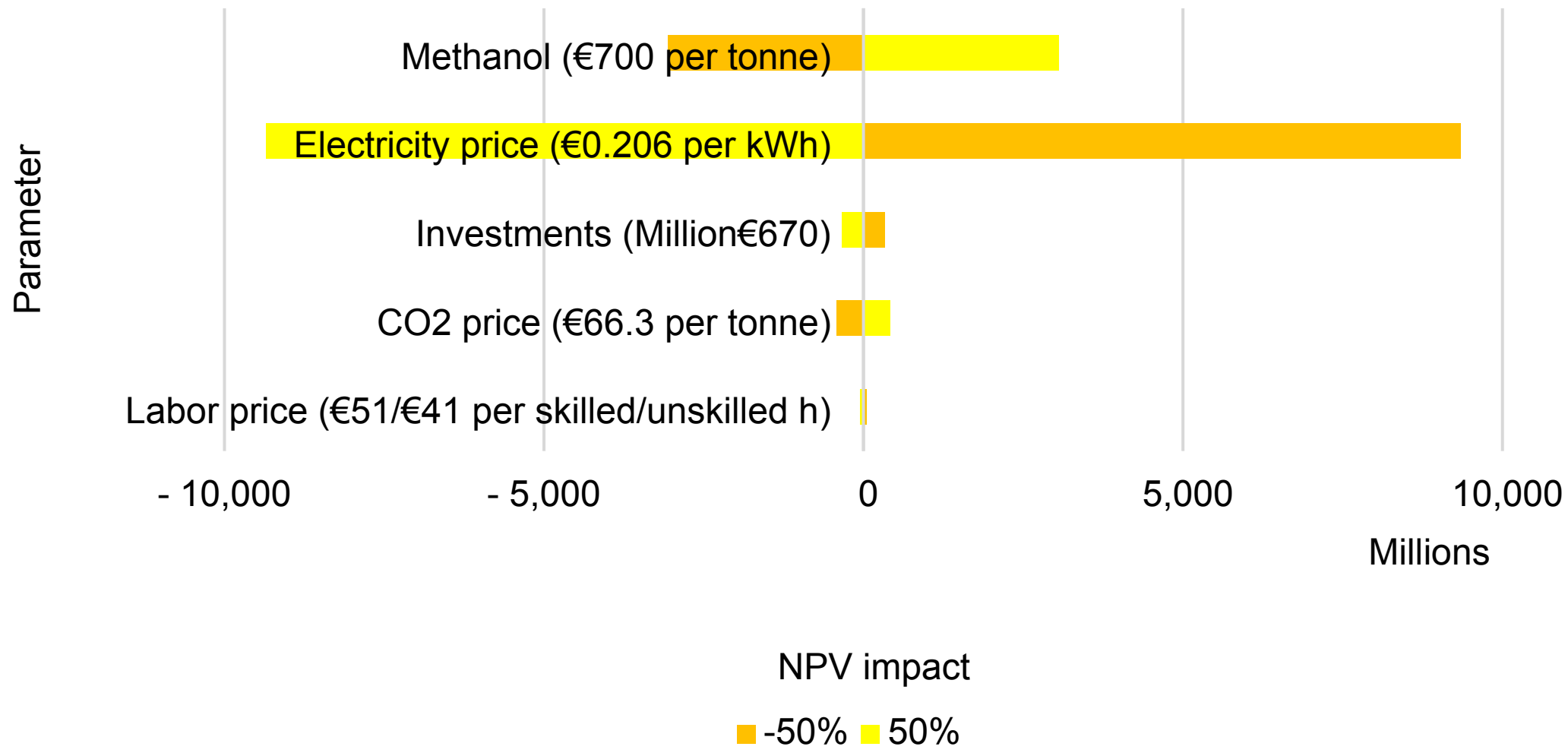

Figure S6: NPV sensitivity analysis results for CO_2 use

References

(1) Voss, R.; Lee, R. P.; Keller, F.; Huang, Q.; Fröhling, M. Decarbonization potential and economic viability of chemical recycling for China's transformation towards carbon neutrality: Case analysis of global warming potential and costs of municipal solid waste gasification for methanol production. *Resources, Conservation and Recycling* **2024**, *206*, 107613. DOI: 10.1016/j.resconrec.2024.107613.

(2) Alvarez, A.; Castañeda, L. C.; Ancheyta, J. On the application of petroleum feedstock modeling techniques for developing molecule-based models of hydrocarbon conversion processes. *Catalysis Today* **2014**, *220-222*, 198–207. DOI: 10.1016/j.cattod.2013.04.009.

(3) Alakangas, E. *Eigenschaften von Biomassebrennstoffen und Vergleich zu fossilen Brennstoffen*, **2005**.

(4) Keller, F. *Life cycle assessment of feedstock recycling processes. Dissertation.* https://nbn-resolving.org/urn:nbn:de:bsz:105-qucosa2-889463 (accessed 2024-02-15).

(5) Krell, K. *Aktualisierung des Sachbilanzmodells des mechanischen Kunststoffrecyclings,* **2023**.

(6) Chemical Engineering. *The chemical engineering plant cost index*. https://www.chemengonline.com/pci-home (accessed 2021-10-13).

(7) Peters, M. S.; Timmerhaus, K. D.; West, R. E. *Plant design and economics for chemical engineers*, 5. ed., internat. ed.; McGraw-Hill chemical engineering series; McGraw-Hill, **2004**.

(8) Zhang, R.; Chen, Y.; Lei, K.; Ye, B.; Cao, J.; Liu, D. Thermodynamic and economic analyses of a novel coal pyrolysis–gasification–combustion staged conversion utilization polygeneration system. *Asia-Pacific J Chem Eng* **2018**, *13* (2). DOI: 10.1002/apj.2171.

(9) Muellerlanger, F.; Tzimas, E.; Kaltschmidt, M.; Peteves, S. Techno-economic assessment of hydrogen production processes for the hydrogen economy for the short and medium term. *International Journal of Hydrogen Energy* **2007**, *32* (16), 3797–3810. DOI: 10.1016/j.ijhydene.2007.05.027.

(10) Pardemann, R. Stoff-Kraft-Kopplung in kohlebasierten Polygenerationskonzepten. Dissertation, Technische Universität Bergakademie Freiberg, **2013**.

(11) Turner, M.; Pinkerton, L. *Quality Guidelines for Energy System Studies: Capital Cost Scaling Methodology.* DOI: 10.2172/1513277, **2013**.

(12) Gräbner, M.; Ogriseck, S.; Ogriseck, K.; Kuske, E.; Abraham, R.; Günster, W.; Metz, T.; Koss, U.; Jensen, S.; Rieger, M.; Wolf, K.-J.; Renzenbrink, W.; Rainer, H.; Folke, C.; Korobov, D.; Beysel, G.; Alekseev, A.; Hannemann, F.; Karkowski, G.; Higman, C.; Morstein, O. von; Rappold, D.; Graeber, C.; Riedl, K.; Kittel, J.; Heil, S.; Rost, M.; Brunhuber, C.; Ulbricht, I.; Worlitz, F.; Rottembach, T.; Rauchfuß, H.; Rochner, I.; Bauersfeld, D.; Trompelt, M.; Guhl, S.; Klinger, M.; Brenner, S.; Meyer, B. *Schlussbericht "Verbundvorhaben COORIVA".* https://edocs.tib.eu/files/e01fb09/60863980X.pdf, **2008** (accessed 2025-08-14).

(13) Goellner, J. F.; Kuehn, N. J.; Shah, V.; White, C. W.; Woods, M. C. *Baseline Analysis of Crude Methanol Production from Coal and Natural Gas (DOE/NETL-341/101514),* **2014**.

(14) Rath, L. *Cost and Performance Baseline for Fossil Energy Plants. Volume 2: Coal to Synthetic Natural Gas and Ammonia (DOE/NETL-2010/1402).* https://www.netl.doe.gov/sites/default/files/netl-file/SNGAmmonia_FR_20110706.pdf., **2011** (accessed 2025-08-14).

(15) Gray, D.; White, C.; Salerno, S.; Plunkett, J.; Tomlinson, G. *Production of High Purity Hydrogen from Domestic Coal: Assessing the Techno-Economic Impact of Emerging Technologies (DOE/NETL-2010/1432).,* **2010**.

(16) Cimpan, C.; Maul, A.; Wenzel, H.; Pretz, T. Techno-economic assessment of central sorting at material recovery facilities – the case of lightweight packaging waste. *J. Cleaner Prod.* **2016**, *112*, 4387–4397. DOI: 10.1016/j.jclepro.2015.09.011.

(17) RECENSO. *Specifications of CTC technology for the study "Waste Utilization Technologies" of TU Freiberg,* **2019**.

(18) Sömnez, H. G. Techno-economic evaluation of a liquid redox process employing amino acid salts for sour natural gas treatment. Dissertation, TU Berlin, **2017**.

(19) Holst, M.; Aschbrenner, S.; Smolinka, T.; Voglstätter, C.; Grimm, G. *Cost forecast for low temperature electrolysis – Technology driven bottom-up prognosis for PEM and alkaline water electrolysis systems.* https://www.ise.fraunhofer.de/content/dam/ise/de/documents/publications/studies/cost-forecast-for-low-temperature-electrolysis.pdf (accessed 2023-04-12).

(20) Wolfersdorf, C. Elektroenergieeinkopplung bei der stofflichen Nutzung von Braunkohle und CO2. Dissertation, TU Bergakademie Freiberg, **2017**.

(21) Voss, R.; Lee, R. P.; Seidl, L.; Keller, F.; Fröhling, M. Global warming potential and economic performance of gasification-based chemical recycling and

incineration pathways for residual municipal solid waste treatment in Germany. *Waste Manage.* **2021**, *134*, 206–219. DOI: 10.1016/j.wasman.2021.07.040.

(22) BfJ. *Körperschaftsteuergesetz*. https://www.gesetze-im-internet.de/kstg_1977/ (accessed 2021-10-13).

(23) BdF. *AfA-Tabelle für die allgemein verwendbaren Anlagegüter*. https://www.bundesfinanzministerium.de/Content/DE/Standardartikel/Themen/Steuern/Weitere_Steuerthemen/Betriebspruefung/AfA-Tabellen/Ergaenzende-AfA-Tabellen/AfA-Tabelle_AV.pdf?__blob=publicationFile&v=3 (accessed 2021-10-13).

(24) DESTATIS. *National Accounts*. https://www.destatis.de/DE/Themen/Wirtschaft/Volkswirtschaftliche-Gesamtrechnungen-Inlandsprodukt/Publikationen/Downloads-Inlandsprodukt/inlandsprodukt-vierteljahr-pdf-2180120.pdf?__blob=publicationFile, **2024** (accessed 2025-02-21).

(25) Yassin, L.; Lettieri, P.; Simons, S. J.R.; Germanà, A. Techno-economic performance of energy-from-waste fluidized bed combustion and gasification processes in the UK context. *Chem. Eng. J.* **2009**, *146* (3), 315–327. DOI: 10.1016/j.cej.2008.06.014.

(26) DESTATIS. *Verbraucherpreisindex (inkl. Veränderungsraten) (61111-0001).* https://www-genesis.destatis.de/genesis/online (accessed 2025-02-21).

(27) IEA. *World Energy Balances*. https://www.iea.org/data-and-statistics/data-product/world-energy-balances, **2024** (accessed 2025-02-21).

(28) Agora Energiewende. *Wie werden Wärmenetze grün?* https://www.agora-energiewende.de/fileadmin/Projekte/2019/Waermenetze/155_Waermenetze_WEB.pdf, **2019** (accessed 2025-02-27).
